# Using transient equilibria (TREQ) to measure the thermodynamics of slowly assembling supramolecular systems

**DOI:** 10.1126/sciadv.abm8455

**Published:** 2022-04-06

**Authors:** Christopher D. Hennecker, Christophe Lachance-Brais, Hanadi Sleiman, Anthony Mittermaier

**Affiliations:** Department of Chemistry, McGill University, 801 Sherbrooke St. W., Montreal H3A 0B8, Canada.

## Abstract

Supramolecular chemistry involves the noncovalent assembly of monomers into materials with unique properties and wide-ranging applications. Thermal analysis is a key analytical tool in this field, as it provides quantitative thermodynamic information on both the structural stability and nature of the underlying molecular interactions. However, there exist many supramolecular systems whose kinetics are so slow that the thermodynamic methods currently applied are unreliable or fail completely. We have developed a simple and rapid spectroscopic method for extracting accurate thermodynamic parameters from these systems. It is based on repeatedly raising and lowering the temperature during assembly and identifying the points of transient equilibrium as they are passed on the up- and down-scans. In a proof-of-principle application to the coassembly of polydeoxyadenosine (polyA) containing 15 adenosines and cyanuric acid (CA), we found that roughly 30% of the CA binding sites on the polyA chains were unoccupied, with implications for high-valence systems.

## Teaser

A method to accurately measure the energies holding together slowly assembling supramolecular polymers is reported.

## INTRODUCTION

Supramolecular chemistry is emerging as a rich source of diverse materials with novel and valuable properties. Potential applications range from drug delivery and tissue regeneration to optical sensors and organic electronics (*1*). This approach involves the noncovalent self-assembly of tens to thousands of monomeric units into larger structures with emergent physical properties that derive from both the structures of the individual components and their interactions and arrangement with respect to one another (*2*). Reversible assembly has some distinct advantages compared with traditional covalent synthesis. The dynamic nature of supramolecular interactions allows bonds to break and reform leading to materials with self-healing properties. Furthermore, many supramolecular systems have the ability to generate multiple morphologies and sets of physical properties from a single set of building blocks with only small modifications of the assembly conditions (*3*). Nevertheless, there are unique challenges associated with this approach. Chief among these is characterizing the products of a noncovalent assembly reaction. Much of the excitement surrounding supramolecular chemistry comes from the fact that there remains much to be understood regarding the relationships between the chemical structures of the monomeric units, the supramolecular architectures, and the emerging physical properties, and there is wide possibility for new and unexpected discoveries. However, this implies that the nature of supramolecular products is difficult to predict and that rigorous structural and thermodynamic analyses are critical to advancing the field.

A variety of tools have been used to elucidate the structures produced by assembly, including atomic force, electron, and super-resolution microscopies, and solid-state nuclear magnetic resonance spectroscopy (*4*–*6*). The stabilities of the assemblies are most commonly measured by thermal analysis. Most supramolecular structures dissociate when they are heated and reassemble when the monomer mixtures are cooled. This process can be quantified either by calorimetry (*7*) or by spectroscopically detected melting and annealing (*8*, *9*). Detailed analyses of melting curves yield the enthalpies, Δ*H*, entropies, Δ*S*, and free energies, Δ*G*, of assembly and shed light on the forces holding the supramolecular structures together (*10*). This information is essential for determining structure/function relationships and the rational design and improvement of self-assembling systems (*11*, *12*). However, there exists a large class of supramolecular systems with extremely slow kinetics that only assemble or disassemble at useful rates when they are pushed far from equilibrium, i.e., under very highly stabilizing or destabilizing conditions. Common examples include amyloid fibrils, viral capsids, and a variety of self-assembling nonbiological small molecules (*11*–*27*). Interest in these kinds of slowly assembling supramolecular systems has grown in recent years, because they allow the size distributions of the resulting fibers to be tightly controlled (*24*, *26*–*28*). Current thermodynamic analyses rely on systems reaching equilibrium before the measurement is taken. In principle, this precludes thermodynamic analyses of slowly assembling systems, because equilibrium is not reached on practical time scales. Nevertheless, it is common practice in the supramolecular field to interpret nonequilibrium thermal data using equations derived for equilibrium systems, despite warnings in the literature that this is invalid (*10*). Our mathematical simulations (see below) indicate that this can lead to errors in reported thermodynamic parameters of >100% and equilibrium constants that differ from their true values by orders of magnitude. Thus, a lack of reliable thermodynamic information for slowly assembling systems is an impediment to the advancement of the supramolecular chemistry field.

We have developed a new experimental approach that can be performed using a standard temperature-controlled spectrophotometer and exploits transient equilibria (TREQ) to provide rigorous thermodynamic data on slowly assembling systems. Rather than waiting for the system to equilibrate (which can take days or weeks), the temperature is repeatedly raised and lowered, driving cyclic, nonequilibrium disassembly and assembly. We find that the system briefly passes through an instant of equilibrium on each up-scan and down-scan at which the rates of assembly and disassembly are equal. The temperatures and concentration values at which these moments of equilibrium occur can be identified from the spectroscopic trace, allowing the full thermodynamic melting curve to be mapped in just a few hours.

**Fig. 1.**
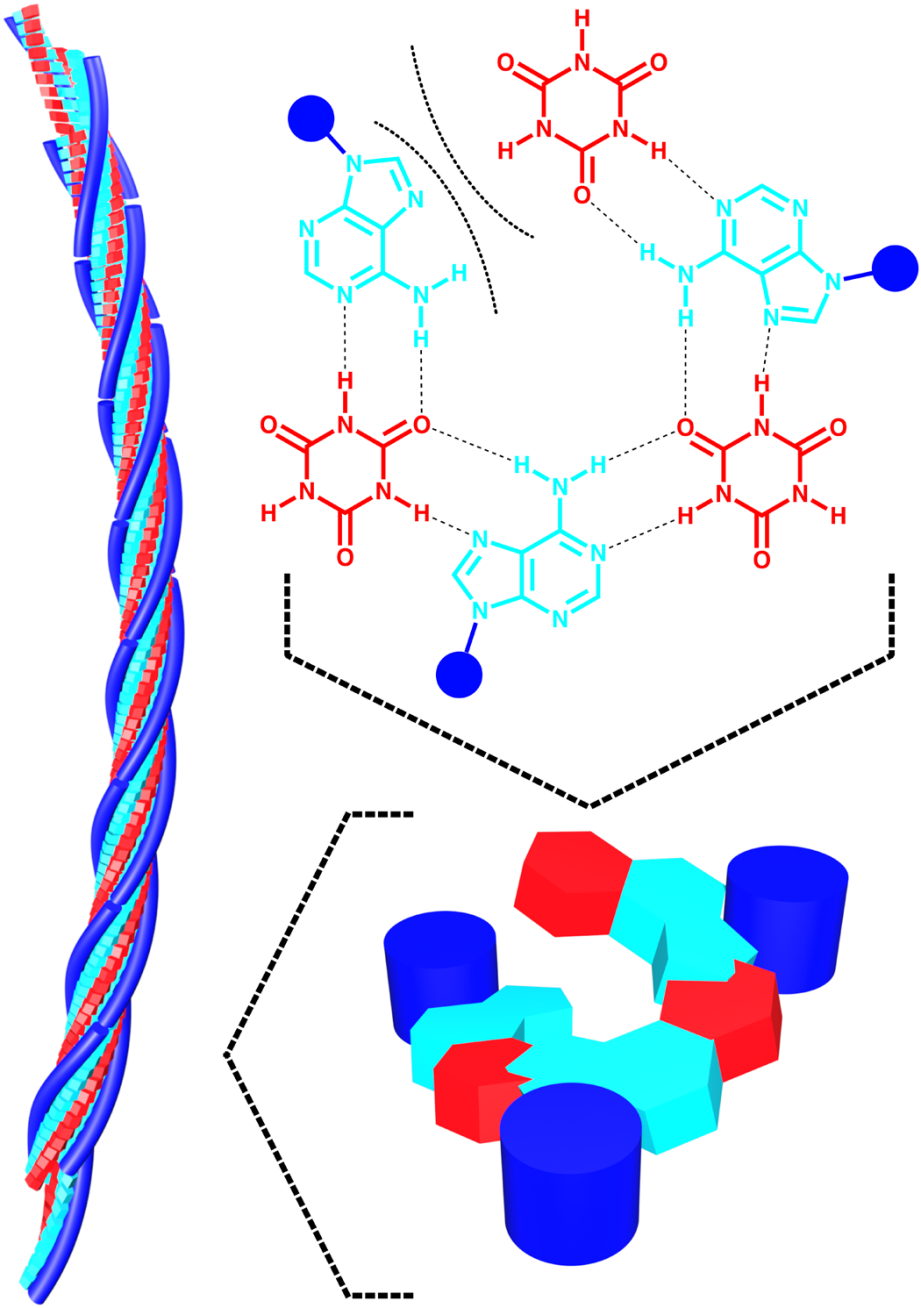
Putative supramolecular structure of polyA-CA fibers. Supramolecular fibers formed from the coassembly of polyadenosine strands and CA (left). A cross section of a single hexameric helicene (right).

As an example, we applied TREQ experiments to better understand the recently found coassembly of polydeoxyadenosine (polyA) and the small-molecule cyanuric acid (CA) into fibers whose biocompatibility and low cost make them promising candidates for tissue engineering and drug delivery (*29*). A cross section of the proposed structure (Fig. 1) shows the deoxy adenosine (dA) of three different DNA strands hydrogen bonding to CA molecules in a continuous supramolecular helicene (*30*, *31*). We note that the ideal helicene structure has a 1:1 ratio of dA residues and CA molecules. We recently characterized the kinetics of polyA-CA fiber assembly using nonequilibrium melting methods (*17*). Equilibration of the fibers near the melting point could take up to a month of constant instrument use. Using TREQ experiments, we determined the Δ*G*, Δ*H*, and Δ*S* values for adding a polyA chain to the end of a growing fiber in a single 10-hour experiment. By repeating these measurements at different concentrations of CA, we determined the minimum polyA:CA ratio necessary for assembly and made the unexpected discovery that about 30% of the available CA binding sites are unfilled under our conditions. These results have implications for the future development of asymmetric systems involving components of very different valences, such as polyA and CA, and demonstrate the potential of the TREQ approach for learning about slowly assembling systems.

## RESULTS AND DISCUSSION

### Theory

**Fig. 2.**
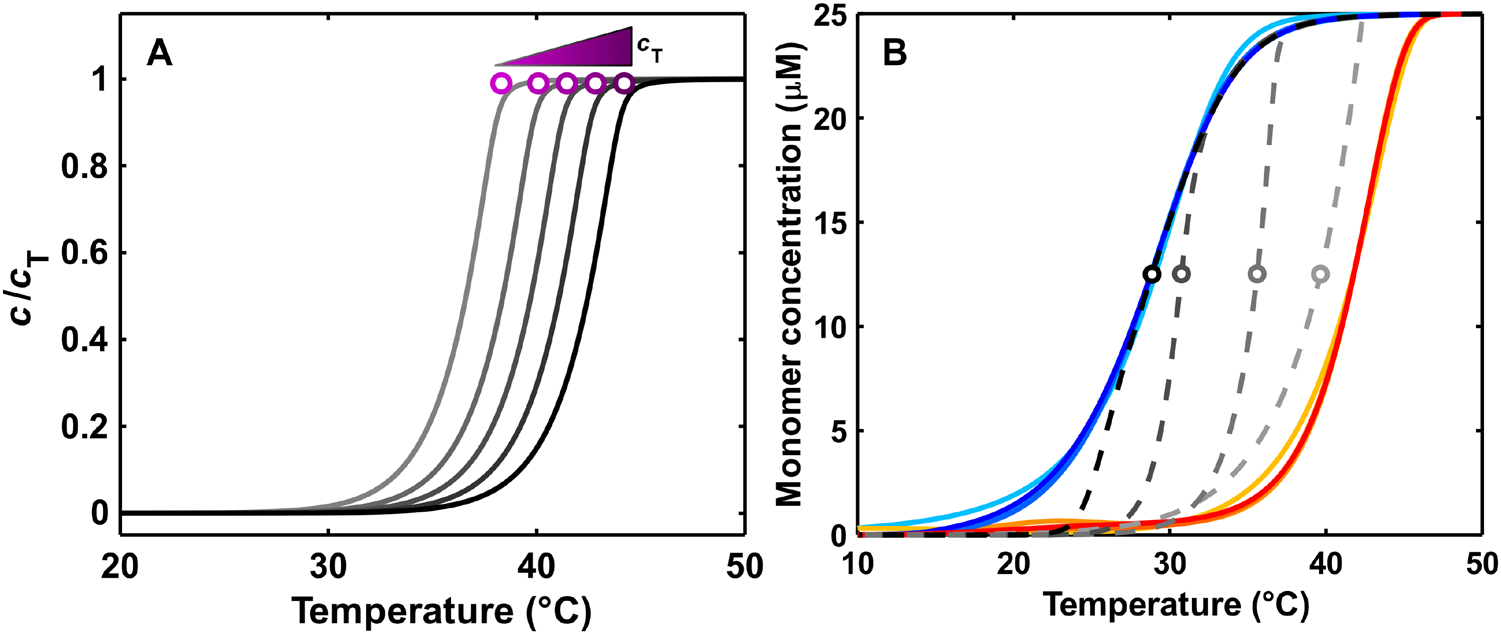
Traditional kinetic and thermodynamic analyses of supramolecular assembly. (**A**) Simulated assembly curves for different total concentrations of monomer (*c*_T_), increasing concentrations are shown as a gradient from gray to black; *T*_e_ values are shown as points using a purple gradient. (**B**) Fiber assembly/disassembly simulated using the GS model and kinetic parameters that give similar melting and annealing curves (solid lines) with drastically different equilibrium curves (dashed lines). Heating curves are shown in red/orange and cooling curves are shown in blue/cyan. The offset between heating and cooling data is due to TH. Simulation parameters are listed in table S3.

Fiber assembly can be described by kinetic schemes such as the Goldstein-Stryer (GS) cooperative kinetic model (*17*, *29*, *32*)
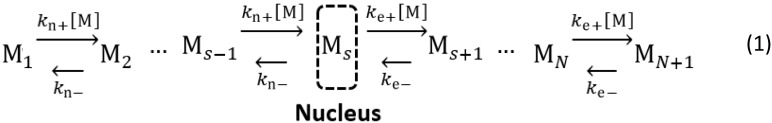
(1)where M*_N_* is a fiber containing *N* monomers. Association and dissociation of monomers from short oligomers less than the critical nucleus size, *s*, are described by the nucleation rate constants *k*_n+_ and *k*_n−_, respectively, while oligomers larger than *s* are described with the elongation rate constants *k*_e+_ and *k*_e−_. An assembly parameter of great importance is the critical monomer concentration, [M]_c_, at which the net rate of assembly or disassembly is zero at equilibrium. For rapidly equilibrating systems, [M]_c_ versus *T* curves can be measured directly by traditional melting or reannealing experiments and analyzed to obtain the enthalpies, entropies, and equilibrium dissociation constants for a monomer adding to the end of a fiber (Δ*H*_e_, Δ*S*_e_, and *K*_e_, respectively) and the corresponding parameters for fiber nucleation (*33*). For cooperative assembly, where nucleation is far less favorable than elongation, [M]_c_ ≈ *K*_e_ and a simplified analysis is commonly used; the maximum temperature at which fibers barely begin to form is identified as the elongation temperature, *T*_e_, and this temperature either can be found by fitting the elongation process or can be approximated from the assembly curve (*34*, *35*), while [M]_c_ is equated to the total monomer concentration, *c*_T_. The experiment is repeated several times at different *c*_T_ values (Fig. 2A), where increasing *c*_T_ leads to an increase in *T*_e_. A van ‘t Hoff plot of ln(*c*_T_) versus 1/*T*_e_ is then used to extract values of Δ*H*_e_ and Δ*S*_e_.

The situation is far more complicated for slowly assembling systems, such as polyA-CA fibers studied here. In these cases, the rate at which the system relaxes to equilibrium is far slower than available temperature scan rates; thus, both folding (cooling) and unfolding (heating) occur out of equilibrium. The populations effectively lag behind the changing temperature such that the cooling and heating scans are offset, in a phenomenon known as thermal hysteresis (TH). Data for the up-scan lie to the right of the equilibrium [M]_c_ versus *T* curve, and data for the down-scan lie to the left, as illustrated in Fig. 2B. The resulting TH loops are rich in kinetic information but are unsuitable for thermodynamic analyses, because the shape and location of the equilibrium curve is ill defined, apart from the fact that it must lie somewhere between the heating and cooling scans (*10*, *17*). To illustrate, fibers obeying the GS assembly model can have very different thermodynamic parameters and equilibrium curves and yet produce nearly superimposable TH data (Fig. 2B).

Nevertheless, data for systems exhibiting pronounced TH have frequently been analyzed as if they were obtained at equilibrium. Heating curves are typically used together with the concentration-dependent *T*_e_ approach described above (*12*, *23*–*25*), although sometimes, cooling scans have been used instead (*11*, *20*–*22*). In their seminal 2003 review, Mergny and Lacroix (*10*) point out that “analysis of the concentration dependency of the denaturation profile only is seriously flawed” and urge “great caution about conclusions reached solely by analysis of the heating curves, a recurrent theme in the literature,” when TH is present. To gain a clearer picture of the magnitude of the problem, we simulated TH data using GS parameters matching our polyA-CA system at different values of *c*_T_ and analyzed the resulting concentration-dependent *T*_e_ values. Using heating scans, the extracted value of Δ*H*_e_ was 2.6-fold too large, whereas using cooling scans, it was 2-fold too small, and *K*_e_ values were incorrect by two to seven orders of magnitude (fig. S1 and table S1). In some studies (*26*, *27*), different temperature scan rates produce superimposable heating data, and it has been argued that this validates their use in the concentration-dependent *T*_e_ analysis. To test this hypothesis, we slightly modified our GS parameters to reproduce this effect and repeated the calculations. The resulting Δ*H*_e_ value was still about 1.8-fold too large (fig. S2 and table S2). Thus, commonly used thermal melting and reannealing experiments do not provide reliable thermodynamic data for slowly assembling systems. Notably, our TREQ method reproduces the thermodynamic parameters in these simulations with a high degree of accuracy (figs. S1 and S2 and tables S1 and S2).

Recent work from the Yamaguchi laboratory (*19*) has explored how the spectra of slowly equilibrating, self-assembling systems respond to repeated heating and cooling cycles (*36*). Depending on the starting and ending temperatures and ramp rates, a rich diversity of shapes (TH loops) have been observed, providing qualitative information on the underlying assembly reactions. However, to date, there has not been a straightforward way to extract quantitative thermodynamic information from these data.

**Fig. 3.**
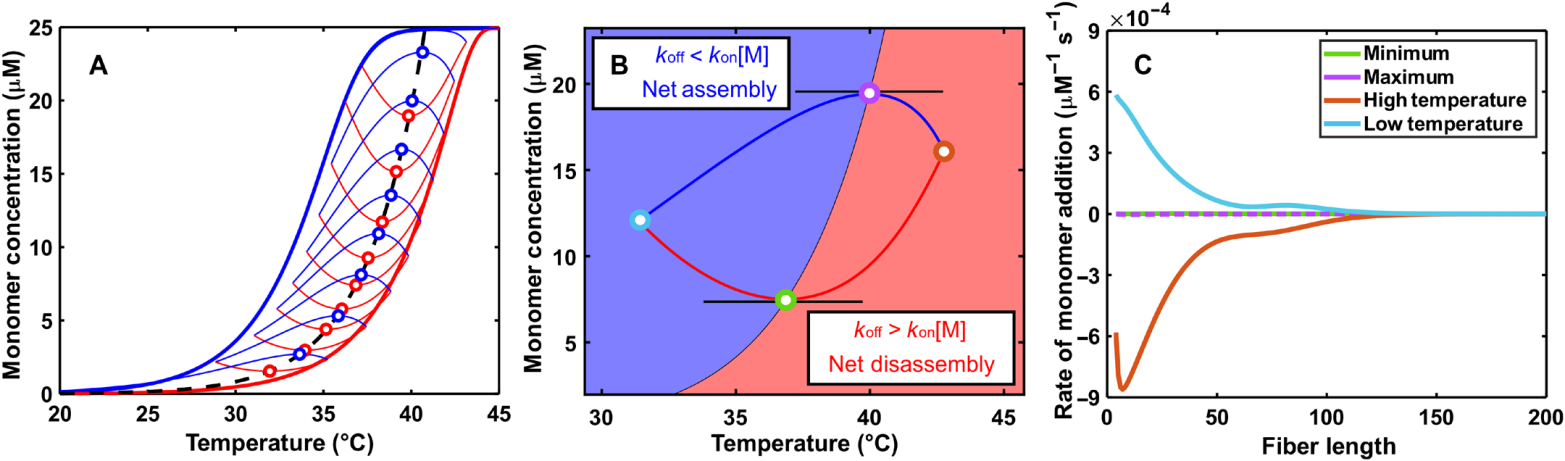
Analysis of a simulated TREQ experiment. (**A**) Kinetic simulations of a typical hysteresis experiment (bold lines) and TREQ experiment (narrow lines). Cooling traces are shown in blue; heating traces are shown in red. The experiment begins by cooling from 45° to 36°C; this is followed by the first up-scan (36° to 44°C), a second down-scan (44° to 35°C), a second up-scan (35° to 43°C), a third down-scan (43° to 34°C), etc. The equilibrium profile is shown as the dashed black line, with the extrema of each TREQ cycle shown as points. (**B**) An isolated TREQ cycle: Assembly occurs only in the blue shaded region; disassembly only occurs in the red shaded region. The interface of these two regions represents a system at equilibrium. Colored points represent the position of calculated monomer flux in (C). (**C**) Calculated monomer flux of fibers for points shown in (B). The horizontal extrema of the TREQ cycle have 100-fold less flux then either the high- or low-temperature values.

Our new TREQ approach uniquely fills this gap. To illustrate the fundamental principles, we performed kinetic simulations using the GS assembly model and parameters for polyA-CA fibers (Fig. 3A and see the Supplementary Materials). The dashed black line indicates the equilibrium [M]_c_ versus *T* curve, while the simulated heating and cooling scans give the left- and right-shifted blue and red curves, respectively. Thus, the location of the true [M]_c_ equilibrium curve is obscured by the TH.

The TREQ method is based on our discovery that repeatedly raising and lowering the temperature such that it repeatedly traverses the equilibrium curve reveals the precise locations of the hidden equilibria. Simulating TREQ data for polyA-CA assembly gives a series of concave-up and concave-down arcs on the heating and cooling scans, respectively (narrow red and blue curves Fig. 3A). Notably, the [M]_c_ values (black line) pass directly through the extrema (concentration maxima and minima) of the cooling and heating arcs. Thus, experimentally determined extrema can be interpreted as a set of [M]_c_(*T*) values. The physical process underlying this behavior can be understood as follows: For cooperatively assembled fibers, such as polyA-CA, equilibrium is reached when the rate of monomer addition to the end of a fiber (*k*_e+_[M]_c_) is exactly equal to the rate of monomer dissociation from the end of a fiber (*k*_e−_), such that the net rate of fiber growth is zero (thus, [M]_c_ ≈ *K*_e_) (*33*). When [M_1_] < [M]_c_, there is net dissociation and [M_1_] increases with time, corresponding to the red region below the [M]_c_ curve in Fig. 3B. When [M_1_] > [M]_c_, there is net association and [M_1_] decreases with time, corresponding to the blue region above the [M]_c_ curve. Every cooling scan starts in the red region with net dissociation (increasing [M_1_]) and ends in the blue region with net association (decreasing [M_1_]). As the temperature crosses the boundary where [M_1_] = [M]_c_, net fiber growth is zero, the arc is exactly horizontal, and the maximum is reached. Conversely, every heating scan starts in the blue region with decreasing [M_1_] and ends in the red region with increasing [M_1_]. As the temperature crosses the [M_1_] = [M]_c_ boundary, the free monomer concentration is at a minimum. To validate this interpretation, we calculated the net rate of monomer addition to each length of fiber in the simulation. At the lower and upper limiting scan temperatures (orange and cyan), the rates of monomer addition and release are at least 100-fold greater than at the horizontal extrema of the heating and cooling arcs (green and purple) (Fig. 3C).

It must be noted that under certain conditions, polyA-CA coassembly can deviate from the GS mechanism depicted in Eq. 1. For example, when polyA chains are mixed with CA at room temperature, fibers grow by a mixture of monomer addition (as described by the GS model) and coagulation (fibers joining end to end) (*37*). The coagulation process introduces structural defects that can be backfilled with additional monomers. In contrast, when free monomers are gradually added to the system over a period of about an hour (through a process of proton dissipation), fibers grow almost exclusively by monomer addition and defects are rare (*37*). Because fiber growth during a TREQ experiment occurs slowly as well, we would expect defects to also be rare in our experiments. In addition, polyA-CA chains are observed to form cable-like structures when formed under proton dissipation conditions (*37*). We note that samples subjected to TREQ heating and cooling cycles do not show evidence of cable formation by atomic force microscopy (fig. S3). Nevertheless, it is worthwhile to discuss the potential effects of such higher-order structures on the TREQ experiment. Cables and other forms of self-association may sequester fiber ends, possibly blocking monomer association and dissociation. However, the termini of the cables are frayed into many individual polyA-CA fibers, where the processes of monomer association and dissociation can be safely assumed to be identical to those in isolated polyA-CA fibers (*37*). The total rates of monomer uptake and release are both directly proportional to the number of exposed fiber ends (*17*, *29*, *32*). Thus, self-association would be expected to alter both rates by the same factor. In contrast, the value of [M]_c_ and the thermodynamics of adding a monomer to a growing fiber do not depend on the number of exposed fiber ends. In the TREQ experiment, the shapes of the heating and cooling arcs depend on the kinetics of polymerization and depolymerization. Slower kinetics due to higher-order structures that sequester fiber ends might be expected to produce flatter arcs. However, the locations of the extrema of the arcs are restricted to lying along the [M]_c_(*T*) curve, which is independent of the number of free ends. Thus, the TREQ experiment is expected to report the thermodynamics of forming individual fibers but does not provide insight into whether or not fibers self-associate or the energetics of such processes.

### Analysis of experimental TREQ data

**Fig. 4.**
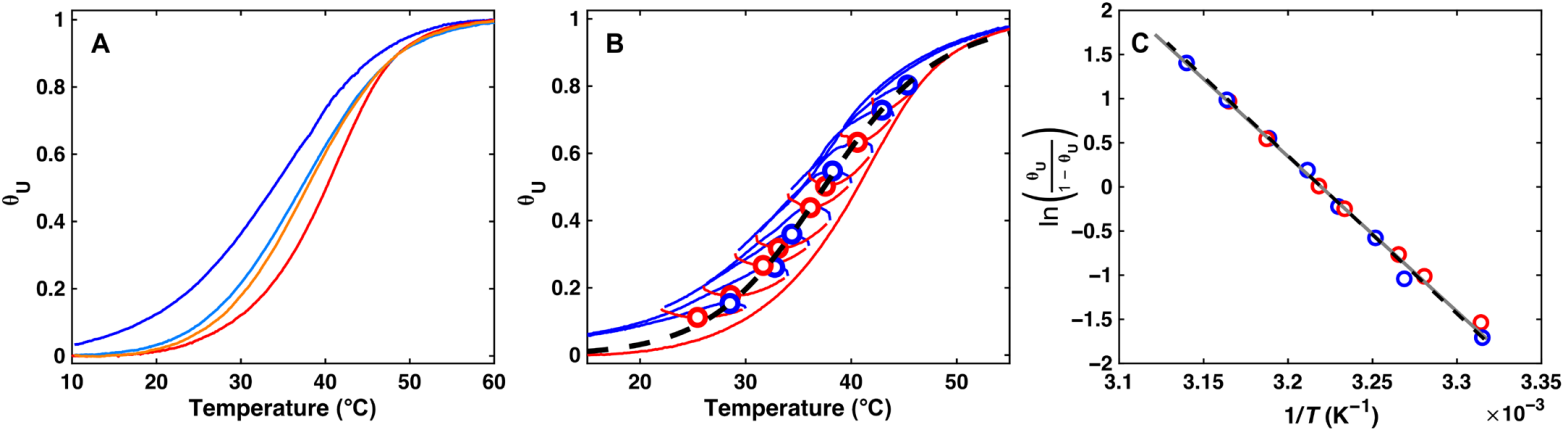
Experimental validation of the TREQ method using an intramolecular G4. (**A**) TH traces of intramolecular G4 folding. Heating and cooling scans at 1 K min^−1^ are shown in red and blue, respectively. Heating and cooling scans at 0.1 K min^−1^ are shown in orange and light blue. (**B**) TREQ data for intramolecular G4 folding. Experimental traces obtained at a scan rate of 0.1 K min^−1^ are shown in blue for cooling and red for heating. Picked extrema are shown as circles, and the equilibrium curve found from analyzing 0.1 K min^−1^ hysteresis traces is shown as the black dashed line. (**C**) Van ‘t Hoff analysis of experimental TREQ points. The line of best fit is shown as the gray solid line, and the equilibrium curve found from analyzing 0.1 K min^−1^ hysteresis traces is shown as the black dashed line.

It is not possible to experimentally confirm that TREQ data follow equilibrium values using the coassembly of polyA and CA as a model system, because the process is so slow that the equilibrium curve is inaccessible to all other experimental techniques that could be used for cross-validation. We therefore turned to a much simpler system, the intramolecular folding of a DNA guanine quadruplex (G4) to experimentally test our approach. G4s are four-stranded, noncanonical nucleic acid structures composed of four tracts of consecutive guanine residues that form stacked, planar, guanine tetrads held together by Hoogsteen hydrogen bonds and coordination to monovalent cations (*38*). Their folding reactions are effectively two-state under many conditions (*38*), and the time scale of folding can be tuned over several orders magnitude simply by adjusting the salt concentration. Heating and cooling scans collected for an intramolecular G4 (see Materials and Methods) with a temperature ramp rate of 1 K min^−1^ are offset by about 6° (Fig. 4A), mimicking the TH observed for polyA-CA, albeit to a lesser extent. In contrast, data for the G4 obtained with a 0.1 K min^−1^ ramp rate are offset by only 0.5°, meaning that they are close to equilibrium during both melting and refolding processes. This small amount of hysteresis, together with the simple folding mechanism, makes it possible to calculate the true equilibrium folding curve with a high level of confidence (*10*). We then performed TREQ analysis on the G4 sample with ±1 K min^−1^ ramp rates, by repeatedly raising and lowering the temperature over a window of roughly 5°C that shifted from (42.3 to 45.7)° to (26.3 to 33.7)°C in 8 cycles while we monitored the spectroscopic absorbance at 295 nm (see the Supplementary Materials for a guide to selecting sliding T-windows). The high- and low-temperature absorbance regions were fitted to linear baselines and assigned 0 and 100% folded, respectively, giving the converted data shown in Fig. 4B. Notably, the experimental equilibrium curve passes nearly exactly through the extrema of the TREQ heating and cooling arcs. Van ‘t Hoff analyses gave Δ*H* = 148 ± 2 kJ mol^−1^ and Δ*S* = 479 ± 6 J mol^−1^ K^−1^ for the equilibrium folding data and Δ*H* = 146 ± 3 kJ mol^−1^ and Δ*S* = 470 ± 10 J mol^−1^ K^−1^ for the TREQ data (Fig. 4C). Thus, the TREQ experiment closely reproduced the results of a traditional equilibrium melting measurement, in a special case where both measurements could be made on the same system.

**Fig. 5.**
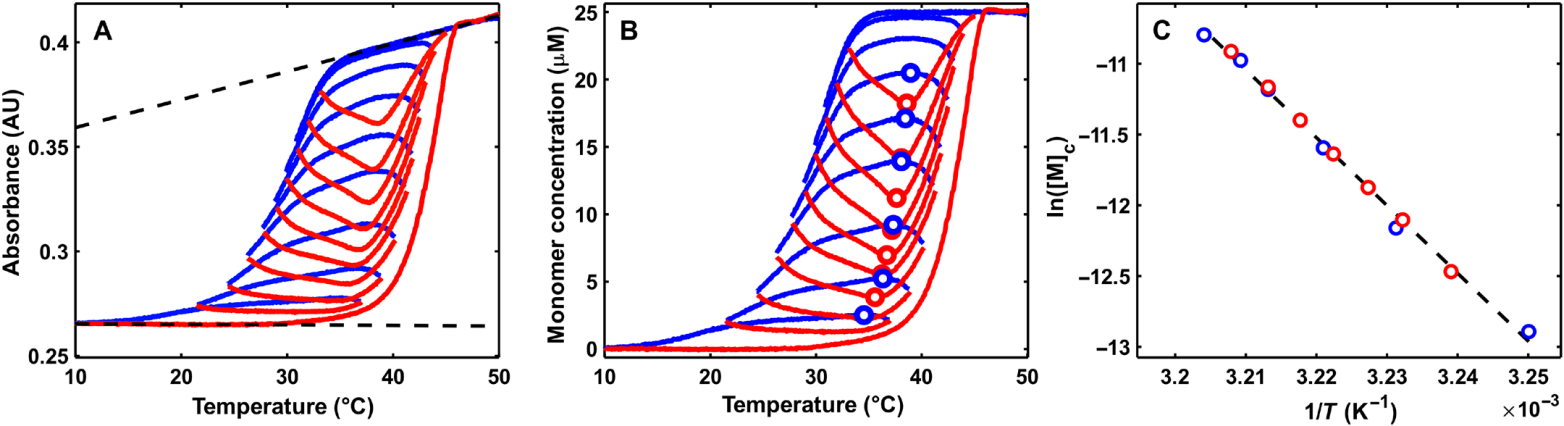
Analysis of TREQ data for polyA-CA coassembly. (**A**) Raw absorbance data for a 15-mer polyA-CA coassembly with 25 μM dA_15_ and 15 mM CA at pH 4.5. Blue lines represent cooling traces and red lines represent heating traces. Unfolded (top black line) and folded (bottom black line) are also shown. AU, arbitrary units. (**B**) TREQ data processed according to eqs. S1 and S2 with extrema of each cycle shown as points. (**C**) Van ‘t Hoff analysis of experimental TREQ points. Line of best fit is shown as the black dashed line.

We then performed a TREQ experiment on a mixture of CA and polyA chains (Fig. 5A). The lower and upper absorbance regions were fitted to linear baselines and assigned 100 and 0% folded, i.e., [M_1_] = 0 and 25 μM, respectively. The fraction of folded monomers at a given temperature was taken as the difference between the measured absorbance and the lower baseline, divided by the difference between the upper and lower baselines (eqs. S1 and S2), as is typically done in spectroscopic analyses of supramolecular assembly (*10*–*12*, *17*, *21*, *23*, *26*, *27*). The converted data are shown in Fig. 5B, with blue and red indicating cooling and heating, respectively, and open circles placed at the extrema. These experimental arcs have a remarkable similarity to the calculations shown in Fig. 3A. The *y* and *x* values of the extrema correspond directly to critical monomer concentration, [M]_c_, and temperature pairs. As discussed above, [M]_c_ values are equivalent to the equilibrium dissociation constant, *K*_e_, for adding a polyA to the end of an elongating fiber, for this system. A van ‘t Hoff plot of ln([M]_c_) = ln(*K*_e_) versus 1/*T* is linear with a slope of −Δ*H*_e_/*R* and *y* intercept of Δ*S*_e_/*R* (Fig. 5C), giving Δ*H*_e_ = 100 ± 2 kcal mol^−1^ and Δ*S*_e_ = 335 ± 7 cal mol^−1^ K^−1^. Notably, although the values of Δ*H*_e_ and Δ*S*_e_ determined by TREQ differ from those obtained by kinetic fits to TH data by factors of 1.6 (table S4), repeating the TH analysis with Δ*H*_e_ and Δ*S*_e_ fixed to the TREQ-derived values gives good agreement with experimental data (fig. S4), illustrating the insensitivity of the kinetic fits to these thermodynamic parameters. In general, we would strongly recommend that even if assembly kinetics are the main interest, the combination of TREQ and TH experiments provide more robust solutions than TH alone, as TREQ resolves ambiguity in the fitted rate constants and ratios thereof.

Furthermore, the thermodynamic parameters provide a basis for comparing polyA-CA fibers to other nucleic acid structures. For example, polyA/polyT (dA_15_dT_15_) duplex dissociation is predicted to have approximately Δ*H* = 108 kcal mol^−1^ and Δ*S* = 335 cal mol^−1^ K^−1^ under similar solution conditions to those used here (*39*). These are intriguingly similar to the values that we measured for polyA-CA assembly (100 kcal mol^−1^ and 335 cal mol^−1^ K^−1^). At first glance, we would have expected polyA-CA fibers to show much higher enthalpies and entropies than dAdT duplexes, because there are three strands rather than two, and each dA forms twice as many hydrogen bonds and immobilizes a CA molecule in the putative polyA-CA structure (Fig. 1). However, partial vacancy of CA binding sites may help to reconcile these observations, as elaborated below.

### Stoichiometry of polyA-CA fibers

One of the great advantages of quantitative thermodynamic data is that much can be learned about the system of interest through careful analyses of how energetic parameters vary with changing conditions. For instance, the presumptive structure of polyA-CA fibers shows that one molecule of CA is present for every deoxyadenosine residue in each polyA chain. In other words, when one of the dA_15_ polyA chains binds the end of an elongating fiber, it should be accompanied by 15 CA molecules. While equilibrium dialysis experiments are consistent with this structure (*29*), they have relatively low precision, and the stoichiometry is very difficult to measure with accuracy. This property is of great interest because a CA:polyA stoichiometry of less than 15 would reveal the existence of defects, which could potentially be targeted with other small molecules. Thermodynamic data can help to resolve this issue, because the apparent dissociation constant, *K*_e_, for a polyA chain binding to the end of the fiber should vary with CA concentration in a predictable way. For instance, if a polyA chain always brings with it *c* molecules of CA, i.e.

$$M_{n}+M_{1}+c\text{CA}\overset{K_{\text{eq}}}{↔}M_{n+1}$$ (2)

(following the nomenclature of Eq. 1), then the full equilibrium dissociation constant for the process is given by

$$K_{\left(T\right)}^{{}^{\circ}}=\frac{[M_{n}][M_{1}]{\left[\text{CA}\right]}^{c}}{\left[M_{n+1}\right]}$$ (3)

This is something of an oversimplification, as elaborated below, but for now, it serves to illustrate the dependence of *K*_e_ on [CA]. For polyA-CA fibers, CA is always in great excess so that its concentration is effectively constant for any set of assembly conditions. The apparent polyA dissociation constant *K*_e_ is related to the full equilibrium constant according to

$$K_{e}={\frac{\left[M_{n}][M_{1}\right]}{\left[M_{n+1}\right]}|}_{\left[\text{CA}\right]}=K_{\left(T\right)}^{{}^{\circ}}{\left[\text{CA}\right]}^{-c}$$ (4)

with the temperature dependence of the standard equilibrium constant $\left(K_{\left(T\right)}^{{}^{\circ}}\right)$ described by

$$K_{\left(T\right)}^{{}^{\circ}}=\text{exp}(-\frac{\left(∆H_{\left(T\right)}-T∆S_{\left(T\right)}\right)}{\text{RT}})$$ (5)

Therefore, measuring *K*_e_ at a series of different CA concentrations should produce offset van ‘t Hoff plots where the vertical distance between each line follows the stoichiometry of CA. To proceed, we noted that stabilization of polyA-CA fibers at high [CA] is largely entropic in nature, because it is primarily driven by differences in the entropy of dilution when dissociation of a polyA chain concomitantly releases *c* molecules of CA into solution.

**Fig. 6.**
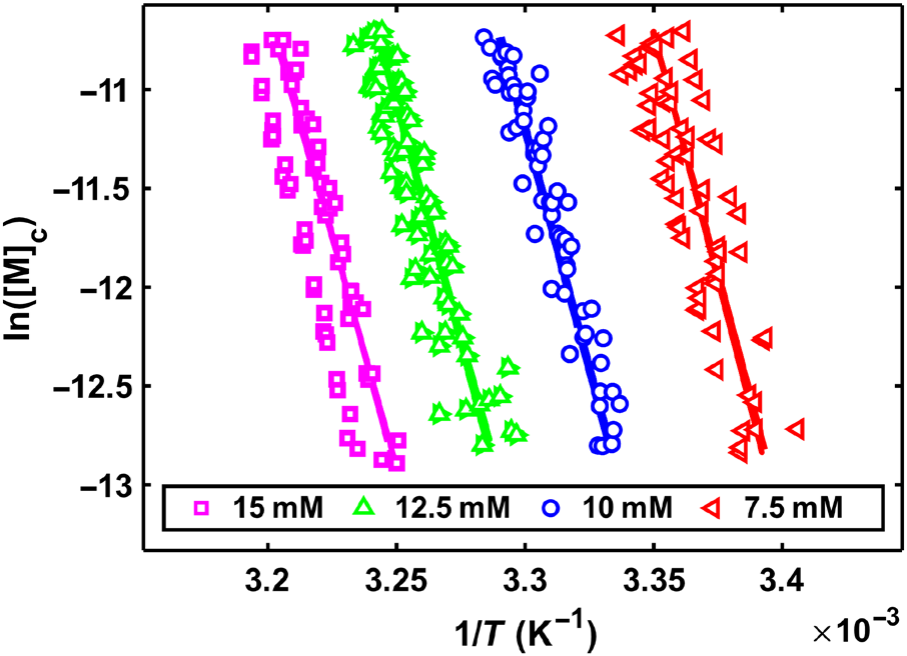
Van ‘t Hoff plot of TREQ data obtained at different CA concentrations. Colored symbols represent experimental data from TREQ traces, solid-colored lines represent a global fit of Eq. 4, and dashed colored lines represent a global fit of Eq. 9. Solid and dashed lines are virtually superimposed on each other. Experimental errors are smaller than the size of the symbols.

We repeated the TREQ experiment at four CA concentrations between 7.5 and 15 mM (fig. S5). Van ‘t Hoff plots of the resulting *K*_e_ values are shown in Fig. 6. Fitting Eq. 4 to this dataset allows us to directly obtain the stoichiometry of CA. To account for the possibility of a temperature-dependent enthalpy value, we extracted global values of Δ*H*_e_ and Δ*C*_p_. The heat capacity change of binding, Δ*C*_p_, accounts for any temperature-dependent differences in the slopes of the different experiments according to

$$∆H_{e}(T)=∆H_{e}(T_{0})+∆C_{p}(T-T_{0})$$ (6)

$$∆S_{e}(T)=∆S_{e}(T_{0})+∆C_{p}\text{ln}(\frac{T}{T_{0}})$$ (7)

The extracted Δ*C*_p_ = −0.6 ± 0.3 kcal mol^−1^ K^−1^ indicates that the enthalpy of adding a polyA chain to a growing fiber has only a slight temperature dependence. This is perhaps expected, because Δ*C*_p_ values associated with nucleic acid folding are largely sequence dependent and have been observed to vary from slightly negative to positive values (*40*). The global fit was in good agreement with experimental data points (Fig. 6 and table S5). Unexpectedly, the extracted stoichiometry coefficient, *c* = 10.4 ± 0.6, implies that 30% of possible CA binding sites are unoccupied in polyA-CA fibers under these conditions.

### Master equations for high-valence assembly

The thermodynamics of multivalent supramolecular assembly can be summarized in terms of two main trends: the “principle of maximum occupancy,” which refers to the tendency of systems to evolve toward the most stable state with full occupancy of binding sites, and the “entropy factor,” which favors the state of the system with the largest number of product species (*41*). For most of the supramolecular systems studied to date, the valency (number of binding sites per monomer) is relatively small (<6), the principle of maximum occupancy dominates, and all sites are generally filled in the assembled materials (*42*, *43*). However, for high-valence monomers, such as the polyA chains studied here, the entropy factor strongly opposes the principle of maximum occupancy and more complex behavior emerges. For example, each dA_15_ chain creates an additional 15 potential CA binding sites, on average, as it adds to the end of growing fiber; one site must be created for each additional dA residue to achieve the theoretical 1:1 dA:CA stoichiometry. The number of ways to fill *c* of the 15 binding sites with *c* molecules of CA is given by the binomial coefficient (*44*)

$$N_{c}=\frac{15!}{c!(15-c)!}$$ (8)

**Fig. 7.**
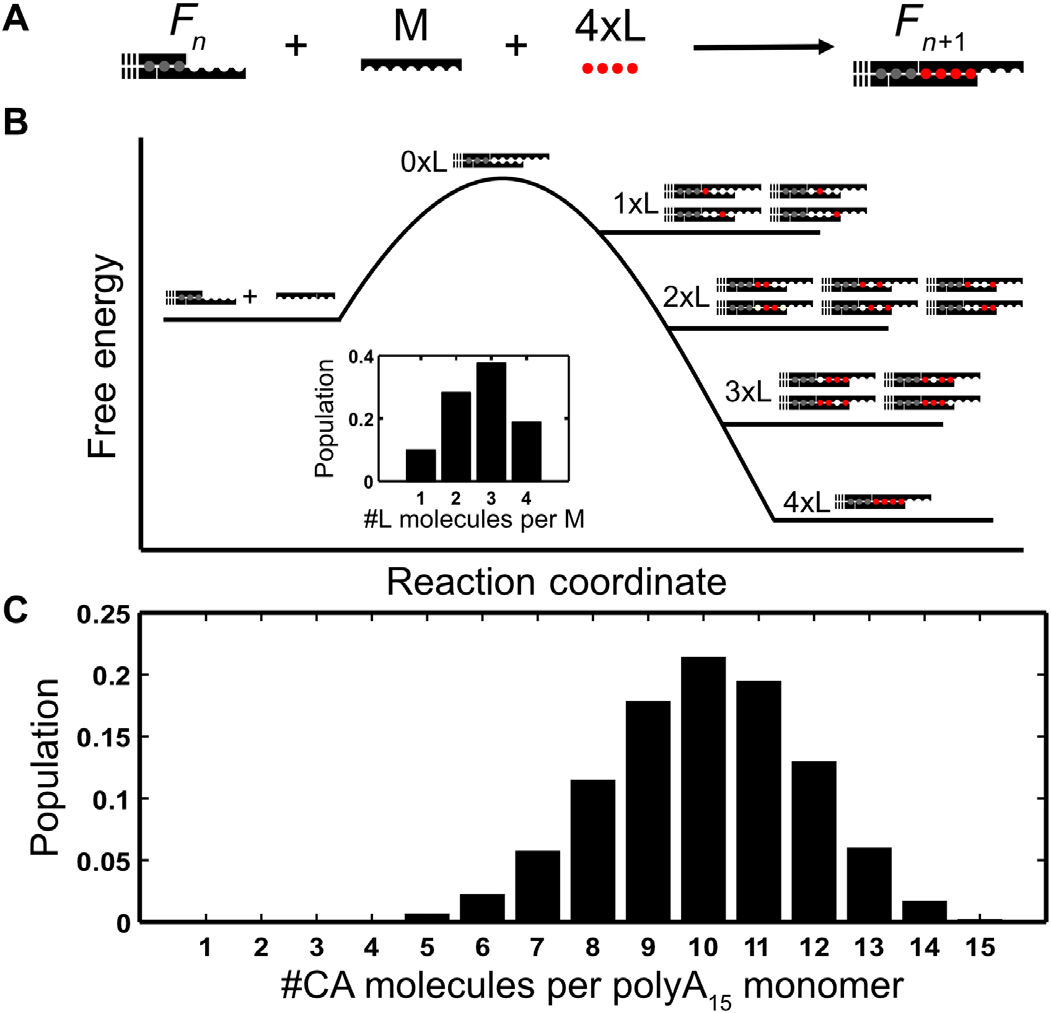
Mechanisms of high-valence assembly. (**A**) A simple constant stoichiometry assembly model where the end of a growing fiber (*F_n_*) assembles with one monomer M and four ligand molecules L to create a fiber of length *n* + 1 (*F*_*n*+1_). (**B**) A free energy diagram of a variable stoichiometry assembly model where the end of a growing fiber (*F_n_*) can assemble with a monomer M and any number of ligand molecules L up to a maximum of 4. The inset represents the populations of each stoichiometry. (**C**) The populations of each stoichiometry for the self-assembly of polyA-CA fibers at 25°C, with a concentration of 12.5 mM CA.

While there is only *N* = 1 way to completely fill all 15 binding sites (*c* = 15), there exists a total of *N* = 32,766 distinct ways to fill the sites with 1 ≤ *c* ≤ 14 molecules of CA. A simplified model of this energy diagram is seen in Fig. 7B, where partially filled states are higher in energy but are more numerous. Therefore, although a polyA chain with 15 bound CA molecules may represent the single lowest-energy configuration, there exists such an enormous number of partly filled configurations that these dominate, with a broad distribution of CA uptake and just 10 of the 15 sites being filled on average as seen in Fig. 7C.

This explanation implies that polyA chains can bring a variable number of CA molecules with them when they attach to the end of a growing fiber, which is inconsistent with Eq. 4, where the stoichiometry is fixed. To resolve this inconsistency, we developed a simple combinatorial model to describe polyA-CA fiber elongation. There is a free energy penalty for bringing an unbound polyA chain in close proximity to the end of a fiber, Δ*G*_polyA_ = Δ*H*_polyA_ − *T*Δ*S*_polyA_. This is compensated by energetically favorable binding of CA molecules to the newly created 15 binding sites. All CA molecules are assumed to bind with equal free energy Δ*G*_CA_ = Δ*H*_CA_ − *T*Δ*S*_CA_. The total free energy change for a polyA chain binding along with a specific configuration of *c* CA molecules is Δ*G*_polyA_ + *c*Δ*G*_CA_. Overall, the apparent equilibrium dissociation constant for polyA chain binding is given by (*45*)

$${\left(K_{e}\right)}^{-1}=K_{\text{polyA}}{\left(1+K_{\text{CA}}[\text{CA}]\right)}^{15}$$ (9)

where *K*_polyA_ = exp(−Δ*G*_polyA_/RT) and *K*_CA_ = exp(−Δ*G*_CA_/RT). The average number of CA molecules can be calculated using the following equation

$$\langle c\rangle =15\frac{K_{\text{CA}}[\text{CA}]}{1+K_{\text{CA}}[\text{CA}]}$$ (10)

and the fraction of bound states with a given number of CA molecules can be calculated by

$$\theta_{c}=(\frac{15!}{c!(15-c)!})\frac{K_{\text{CA}}{\left[\text{CA}\right]}^{c}}{{\left(1+K_{\text{CA}}[\text{CA}]\right)}^{15}}$$ (11)

We fit Eq. 9 to the TREQ data, obtaining excellent agreement, and extracting Δ*H*_polyA_, Δ*S*_polyA_, Δ*H*_CA_, and Δ*S*_CA_ (Fig. 6 and table S6). These parameters allowed us to calculate the fractions of polyA chains with different numbers of CA molecules bound at different temperatures and [CA], providing a highly detailed description of assembly (Fig. 7C). Under highly stabilizing conditions of high [CA] and low temperature, the equations predict that almost all binding sites are filled, in agreement with previous dialysis experiments (*29*). Equations 9 and 10 explain why we observe 10 bound CA, and not more or less, even though experiments were performed at different [CA]. All experiments used 25 μM polyA, which means that we only detected *K*_e_ values between about 3 and 22 μM in all cases. This implies that the *K*_CA_[CA] values are nearly identical in all experiments (because *K*_polyA_ does not change much with temperature). From Eq. 10, this implies that ⟨*c*⟩ is very similar in all experiments, ranging from 10 to 11, and in excellent agreement with the simple fit described in the previous section.

High-valence supramolecular systems have many useful properties that are only just beginning to be explored, such as the ability to self-heal, responsiveness to stimuli, and simple, inexpensive chemical derivatization. Examples include small molecule–directed nucleic acid assembly [CA + polyadenosine or polyA (*17*, *29*); melamine + polythymine (*46*)] and noncovalent polymer cross-linking via multiple metal chelation (*42*, *47*) or host/guest interactions (*48*, *49*). Equations 9 and 10 can serve as starting points for quantitatively describing assembly in such systems, where simple probabilistic considerations ensure that some of the binding sites will remain vacant under many conditions. Furthermore, we find that TREQ-derived data are sufficient to extract the relevant thermodynamic parameters robustly, providing a new avenue for gaining insight into these complex materials.

### Generality of the method

Our aim for the TREQ method is that it can be used as a general tool to determine the thermodynamic parameters of supramolecular assembly when standard thermal melting and annealing experiments are unsuitable for thermodynamic analysis. Toward this end, we have also tested the method on a tetrameric intermolecular G4 in aqueous buffer and zinc-porphyrin self-assembly in mixture of methylcyclohexane and chloroform. In both cases, we obtained series of concave-up and concave-down arcs, similar to those of the polyA-CA fibers (fig. S9). In parallel, we used computer simulations to model the TREQ experiment for different types of self-assembling systems and observed two patterns of behavior: either all the extrema aligned with the equilibrium curve or the maxima for the cooling curves and minima for the heating curves were offset from one another (fig. S9). This provides a useful guide for interpreting TREQ data on new systems of interest: When the extrema align, they can be used to trace out the equilibrium curve (as for polyA-CA fibers and the intermolecular G4). When they are offset, they cannot be directly equated to equilibrium temperature/concentration pairs (as for the zinc porphyrin system), although the data are still information rich, as detailed in the Supplementary Materials. Fortunately, many slowly assembling supramolecular structures are amenable to the TREQ approach and, in these cases, it provides thermodynamic information that is not readily available from other sources. For example, a polyA-CA [M]_c_ dataset similar to the one reported here would require a scan rate of <0.001 K min^−1^ in traditional melting measurements, leading to experiments on the impractically long time scale of a month. Our study demonstrates how the ready availability of high-quality thermodynamic dynamic data can lead to new insights, such as the prevalence of unfilled CA binding sites in polyA-CA fibers, and provides an opportunity to test theoretical developments, such as our master equation for high-valence assembly. These advances would not have been realistically possible for polyA-CA structures using previously existing methods.

A large number of slowly assembling supramolecular systems have been described in the literature, with only a subset referenced in this study (*11*–*27*). This field is expected to expand in the coming years, because slow, nonequilibrium, nucleated assembly is a living polymerization process. The advantages of living polymers in supramolecular chemistry are an area of active research, with benefits already evident in the level of control that they give over fiber length and monodispersity (*24*, *26*–*28*). Notably, thermodynamic information for slowly assembling systems is either completely lacking or determined using methods that we and others (*10*) have shown to be unreliable for these systems. We believe that the TREQ method presented here is a big step toward filling this gap in our knowledge. It can be applied to a wide variety of systems using common benchtop laboratory equipment, and measurement times are on the order of 10 hours. The experiments are straightforward to set up, and a typical analysis (e.g., van ‘t Hoff plot) can be performed entirely using standard spreadsheet software (see the Supplementary Materials). We believe that the TREQ method will prove generally useful to the supramolecular chemistry community.

## MATERIALS AND METHODS

### Materials

#### Intramolecular G4

A 22-mer mutant of the cMYC G4 (TGAGGGTIGGGAGGGTGGGIAA) was synthesized using a MerMade-12 Oligonucleotide Synthesizer with standard solid-phase phosphoramidite chemistry (*18*). The G4 samples were cartridge purified and analyzed by liquid chromatography–mass spectrometry for purity. DNA strands were dissolved in MilliQ water and concentrations were calculated using nearest-neighbor extinction coefficients. Buffer consisted of 10 mM lithium phosphate (pH 7.0) supplemented with 250 μM KCl. The buffer pH was titrated using 1 M LiOH to avoid the further addition of stabilizing Na^+^ or K^+^ cations.

#### Polydeoxyadenosine–cyanuric acid

CA, tris, magnesium chloride hexahydrate (MgCl_2_·6H_2_O), sodium chloride (NaCl), glacial acetic acid, and urea were used as purchased from Sigma-Aldrich. Boric acid was obtained from Thermo Fisher Scientific and used as supplied. Acrylamide/bis-acrylamide (40% 19:1) solution, ammonium persulfate, and tetramethylethylenediamine were used as purchased from BioShop Canada Inc.

d(A_15_) oligonucleotides were synthesized on a MerMade-12 synthesizer, purified by denaturing polyacrylamide gel electrophoresis (PAGE; 20%, 1× tris-borate EDTA running buffer, and 8 M urea) and desalted with Gel-Pak desalting columns from Glen Research. Purity of the strand was confirmed by high resolution mass spectrometry (calculated mass, 4635.18; observed mass, 4634.28).

Stock solutions of 20 mM CA were prepared by dissolution in 100 ml of Milli-Q water in a volumetric flask and adjusted with acetic acid to pH 4.5. To properly dissolve and degas the solutions, they were heated at 65°C and sonicated and then cooled down to room temperature before being used.

Samples of 100 μl of dA_15_ (25 μM) and CA (7.5, 10.0, 12.5, and 15.0 mM) in pH 4.5 Mg(OAc)_2_ buffer (7.6 mM) were made in quadruplicates. A thin layer (~30 μl) of silicon oil was applied on top to prevent evaporation during experiments.

### Instrumentation

#### Intramolecular G4

Ultraviolet-visible (UV-vis) absorbance studies were performed using a 10-mm quartz cuvette with a 3-mm aperture and monitored at 295 nm on an Agilent Cary 3500 Series UV-vis spectrophotometer equipped with a Peltier temperature controller and in-cell thermal probe. A TH scan was performed from 60° to 10°C at 1 and 0.1 K min^−1^, with an equilibration time of 30 min at both high and low temperatures. TREQ experiments were ran at 1 K min^−1^ with temperature windows chosen from the TH scans. The maximum number of scans on the Cary 3500 is 10, so two TREQ experiments were performed and combined to create Fig. 4 (B and C).

#### Polydeoxyadenosine–cyanuric acid

UV-vis absorbance–based quantification of d(A_15_) was performed on a Nanodrop Lite spectrophotometer from Thermo Fisher Scientific. DNA purification by PAGE was carried out on a 20 cm by 20 cm vertical acrylamide Hoefer 600 electrophoresis unit.

UV-vis absorbance studies were performed using a 1.0-mm quartz cuvette and monitored at 260 nm on an Agilent Cary 300 Series UV-vis spectrophotometer equipped with a Peltier temperature controller and water recirculator. A variable temperature range, which started from 50° to 40°C and went down to 10° to 4°C, was scanned at a rate of 0.5°C/min and with an equilibration time of 30 min at the maximum and minimum temperatures. Argon gas and drierite were used to dry the chamber at temperatures below 10°C.

Atomic force microscopy (AFM) imaging was effectuated on polyA-CA fibers resulting from different annealing procedures. The samples of dA_15_ polyA were composed as described in the “Materials” section, with 12.5 mM CA. The isothermal sample was left at room temperature for 1 day. The thermally annealed sample was annealed at a rate of 0.5°C/min from 50° to 8°C with an initial hold of 30 min at the highest temperature. The TREQ sample was initially heated 50°C for 30 min and then cycled through the TREQ points as described for that CA concentration. For all samples, 5 μl was pipetted onto a freshly cleaved mica surface for 30 s, followed by wicking off the liquid from the surface with a filter paper. The surface is additionally dried with a stream of compressed air for 30 s before being put under vacuum for at least 2 hours before imaging. AFM images were collected on a Multimode 8 scanning probe microscope from Bruker with a Nanoscope V controller equipped with a ScanAsyst-Air silicon tip on nitride lever (tip radius, 2 nm; *k* = 0.4 N/m; and fo = 70 kHz; Bruker).

## Supplementary Materials

### This PDF file includes:

Figs. S1 to S9 Tables S1 to S7 Converting absorbance data to concentrations Guide for acquisition of TREQ data Guide for processing TREQ data Thermodynamic analysis Simplified analysis Generality of the method TREQ simulations References
