# Supplementary material for: Using transient equilibria (TREQ) to measure the thermodynamics of slowly assembling supramolecular systems
Source: Sci Adv. 2022 Apr 6;8(14):eabm8455. doi: 10.1126/sciadv.abm8455 (PMC8985918; doi:10.1126/sciadv.abm8455)
Supplement: Supplementary file 1 — Figs. S1 to S9 Tables S1 to S7 Converting absorbance data to concentrations Guide for acquisition of TREQ data Guide for processing TREQ data Thermodynamic analysis Simplified analysis Generality of the method TREQ simulations References [file sciadv.abm8455_sm.pdf]

Science Advances
AAAS

Supplementary Materials for

Using transient equilibria (TREQ) to measure the thermodynamics of slowly assembling supramolecular systems

Christopher D. Hennecker, Christophe Lachance-Brais, Hanadi Sleiman, Anthony Mittermaier*

*Corresponding author. Email: anthony.mittermaier@mcgill.ca

Published 6 April 2022, *Sci. Adv.* **8**, eabm8455 (2022)
DOI: 10.1126/sciadv.abm8455

This PDF file includes:

Figs. S1 to S9
Tables S1 to S7
Converting absorbance data to concentrations
Guide for acquisition of TREQ data
Guide for processing TREQ data
Thermodynamic analysis
Simplified analysis
Generality of the method
TREQ simulations
References

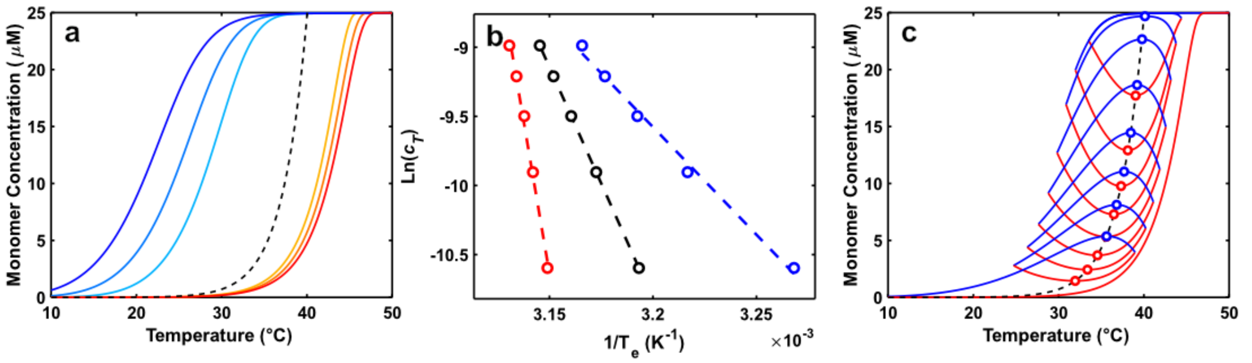

Figure S1: ***Thermodynamic analysis of simulated data.*** *a) Simulated TH traces at 0.5, 1, and 2°C/min scan rates, showing hysteresis in both the heating and cooling traces. Cooling traces are shown as a cyan-blue gradient, heating traces are shown as an orange-red gradient, the true equilibrium trace is shown as the black dashed line. b) TREQ analysis of the heating (red), cooling (blue) and equilibrium (black) curves at 25, 50, 75, 100, 125 uM total monomer concentration. The heating and cooling curves ran at 0.5°C/min. c) Simulated TREQ Analysis ran at 0.5°C/min, cooling traces are shown in blue and heating traces are shown in red. Extrema from each trace are shown as dots, and the true equilibrium is shown as a dashed black line.*

**Activation Energies**		**Rate constants**		**Thermodynamic Constants**		**T_e Analysis Cooling**	**T_e Analysis Heating**	**TREQ Analysis**
E_{n+}	-14	k_{n+}	2.6 x 10^6	ΔG_e	-16	-12	-28	-16
E_{n-}	11	k_{n-}	5.9 x 10^3	ΔH_e	67	31	175	66
E_{e+}	-9	k_{e+}	1.7 x 10^6	ΔS_e	193	80	532	191
E_{e-}	58	k_{e-}	2.0 x 10^{-1}	K_e	1.2e-7	6.2e-6	3.5e-13	1.1e-7

Table S1: ***Kinetic and thermodynamic parameters used to simulate the data in Figure S1.*** *Thermodynamic parameters found from the common TREQ analysis of the cooling curves and heating curves, as well as the thermodynamic parameters found from TREQ analysis. TREQ values were found by fitting the elongation region of the transition to the model developed by Meijer and coworkers.(34, 35) E_{n+}, E_{n-}, E_{e+}, E_{e-}, ΔG_e and ΔH_e values are reported in kcal mol^{-1}, k_{n+} and k_{e+} are reported in M^{-1} min^{-1}, k_{n-}, and k_{e-} are reported in min^{-1}, ΔS_e is reported in cal mol^{-1} K^{-1}, K_e values are reported in M. Rate constants, equilibrium constants, and ΔG_e are reported at a reference temperature of 25˚C.*

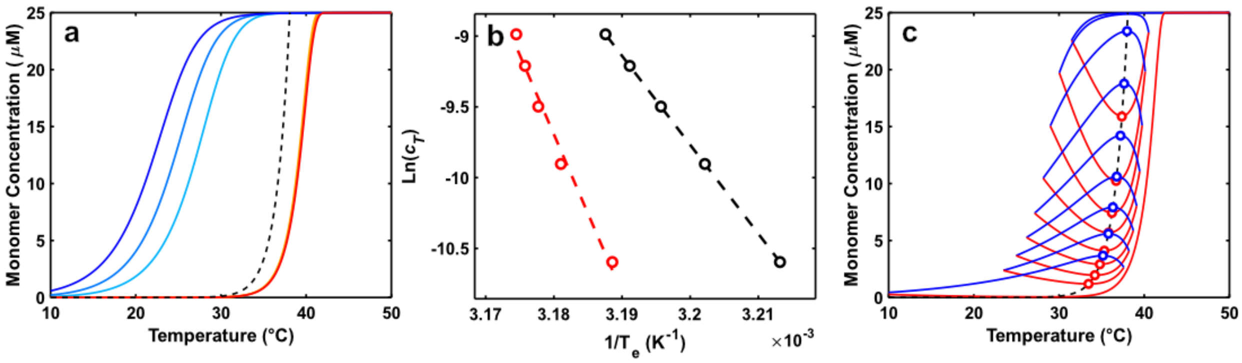

Figure S2: ***Thermodynamic analysis of simulated data with no hysteresis observed during heating.*** *a) Simulated TH traces at 0.5, 1, and 2°C/min scan rates, showing hysteresis in only the cooling traces. Cooling traces are shown as a cyan-blue gradient, heating traces are shown as an orange-red gradient, the true equilibrium trace is shown as the black dashed line. b) TREQ analysis of the heating (red), and equilibrium (black) curves at 25, 50, 75, 100, 125 uM total monomer concentration. The heating and cooling curves ran at 0.5°C/min. c) Simulated TREQ Analysis ran at 0.5°C/min, cooling traces are shown in blue and heating traces are shown in red. Extrema from each trace are shown as dots, and the true equilibrium is shown as a dashed black line.*

**Activation Energies**		**Rate constants**		**Thermodynamic Constants**		**T_e Analysis Heating**	**TREQ Analysis**
E_{n+}	5	k_{n+}	1.5×10^5	ΔG_e	-19	-29	-19
E_{n-}	53	k_{n-}	1.5×10^2	ΔH_e	125	222	125
E_{e+}	-2	k_{e+}	9.8×10^5	ΔS_e	380	688	380
E_{e-}	123	k_{e-}	3.5×10^{-3}	K_e	3.6e-9	2.1e-13	3.6e-9

Table S2: ***Kinetic and thermodynamic parameters used to simulate the data in Figure S2.*** *Thermodynamic parameters found from the common TREQ analysis of the heating curves, as well as the thermodynamic parameters found from TREQ analysis. TREQ values were found by fitting the elongation region of the transition to the model developed by Meijer and coworkers.(34, 35) E_{n+}, E_{n-}, E_{e+}, E_{e-}, ΔG_e and ΔH_e values are reported in kcal mol^{-1}, k_{n+} and k_{e+} are reported in M^{-1} min^{-1}, k_{n-}, and k_{e-} are reported in min^{-1}, ΔS_e is reported in cal mol^{-1} K^{-1}, K_e values are reported in M. Rate constants, equilibrium constants, and ΔG_e are reported at a reference temperature of 25˚C.*

**ΔH_e = 50 : ΔS_e = 138**				**ΔH_e = 100 : ΔS_e = 301**			
**Activation Energies**		**Rate constants**		**Activation energies**		**Rate constants**	
E_{n+}	-1.0	k_{n+}	2.9×10^5	E_{n+}	0	k_{n+}	9.2×10^5
E_{n-}	53	k_{n-}	54	E_{n-}	74	k_{n-}	8.6
E_{e+}	7.5	k_{e+}	3.1×10^5	E_{e+}	-30	k_{e+}	2.1×10^4
E_{e-}	58.5	k_{e-}	7.4×10^{-2}	E_{e-}	70	k_{e-}	8.1×10^{-4}
**ΔH_e = 150 : ΔS_e = 472**				**ΔH_e = 200 : ΔS_e = 644**			
**Activation energies**		**Rate constants**		**Activation energies**		**Rate constants**	
E_{n+}	0	k_{n+}	3.4×10^6	E_{n+}	0	k_{n+}	8.5×10^6
E_{n-}	81	k_{n-}	23	E_{n-}	78	k_{n-}	60
E_{e+}	-82	k_{e+}	5.9×10^3	E_{e+}	-129	k_{e+}	2.7×10^2
E_{e-}	68	k_{e-}	6.8×10^{-4}	E_{e-}	71	k_{e-}	5.1×10^{-4}

Table S3: ***Kinetic parameters for each TH simulation in Figure 2a in the main text****. Activation energies are given in kcal mol^{-1} rate constants are in M^{-1} min^{-1} and min^{-1} for forward and reverse steps respectively and reported at a reference temperature of 25˚C. ΔH_e values are given in kcal mol^{-1} and ΔS_e values are given in cal mol^{-1} K^{-1}.*

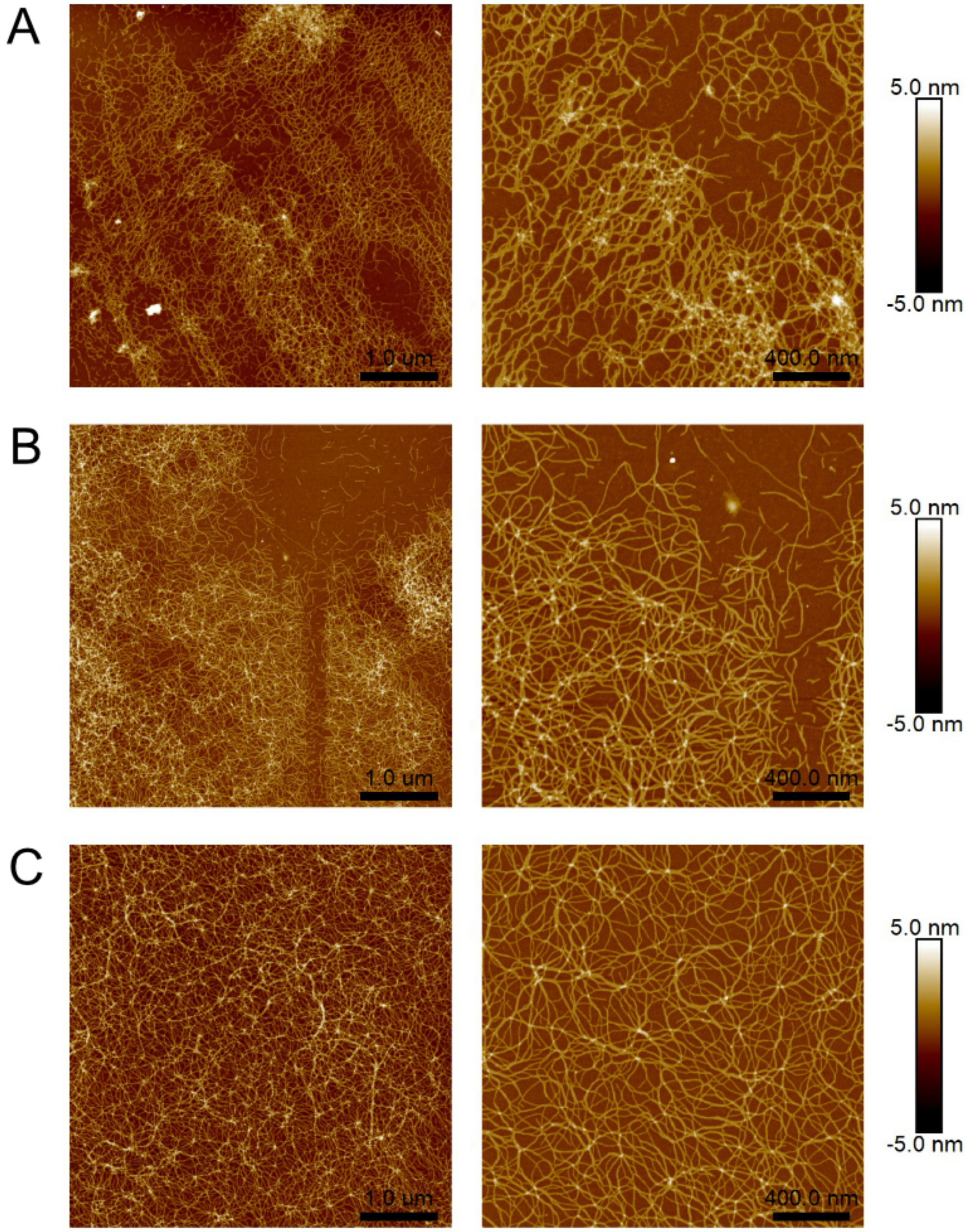

Figure S3: ***AFM images collected in air of dA15 polyA-CA fibres*** *obtained from a) isothermal annealing, b) thermal annealing, and c) TREQ annealing. In all cases fibre formation is seen with no larger cable-like structures observed.*

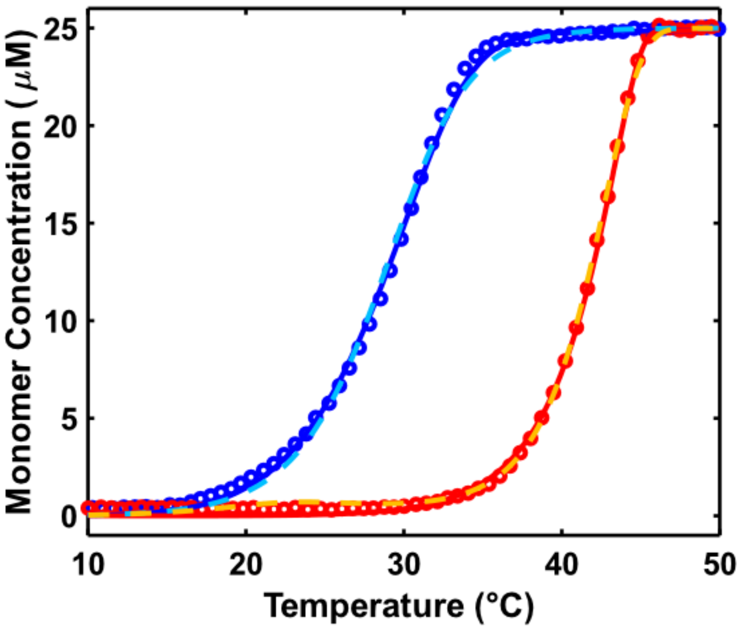

Figure S4: ***Fits of experimental TH curves.*** *With ΔH_e extracted from TH data alone (blue and red solid lines) and ΔH_e constrained to be equal to the value extracted from TREQ measurements (dashed cyan and orange lines). Simulation parameters are listed in Table S4.*

**Unconstrained Parameters**<br><br>**ΔH_e = 62 : ΔS_e = 197**				**Constrained Parameters**<br><br>**ΔH_e = 100 : ΔS_e = 335**			
**Activation energies**		**Rate constants**		**Activation energies**		**Rate constants**	
E_{n+}	-24	k_{n+}	7.6 x 10^{6}	E_{n+}	-13	k_{n+}	5.2 x 10^{6}
E_{n-}	-5	k_{n-}	8.7 x 10^{3}	E_{n-}	62	k_{n-}	46
E_{e+}	-9	k_{e+}	6.9 x 10^{5}	E_{e+}	-31	k_{e+}	1.7 x 10^{4}
E_{e-}	53	k_{e-}	1.2 x 10^{-1}	E_{e-}	69	k_{e-}	7.5 x 10^{-4}
RSS	2.6 x 10^{-4}	T_{ref}	25	*RSS*	3.7 x 10^{-4}	T_{ref}	25

Table S4: ***Kinetic parameters for each TH fit in Figure S4****. Activation energies are given in kcal mol^{-1} rate constants are in M^{-1} min^{-1} and min^{-1} for forward and reverse steps respectively. ΔH_e values are given in kcal mol^{-1}, ΔS_e values are given in cal mol^{-1} K^{-1} and ΔCp values are given in kcal mol^{-1} K^{-1} in the constrained fit, a CA concentration of 15mM was used.*

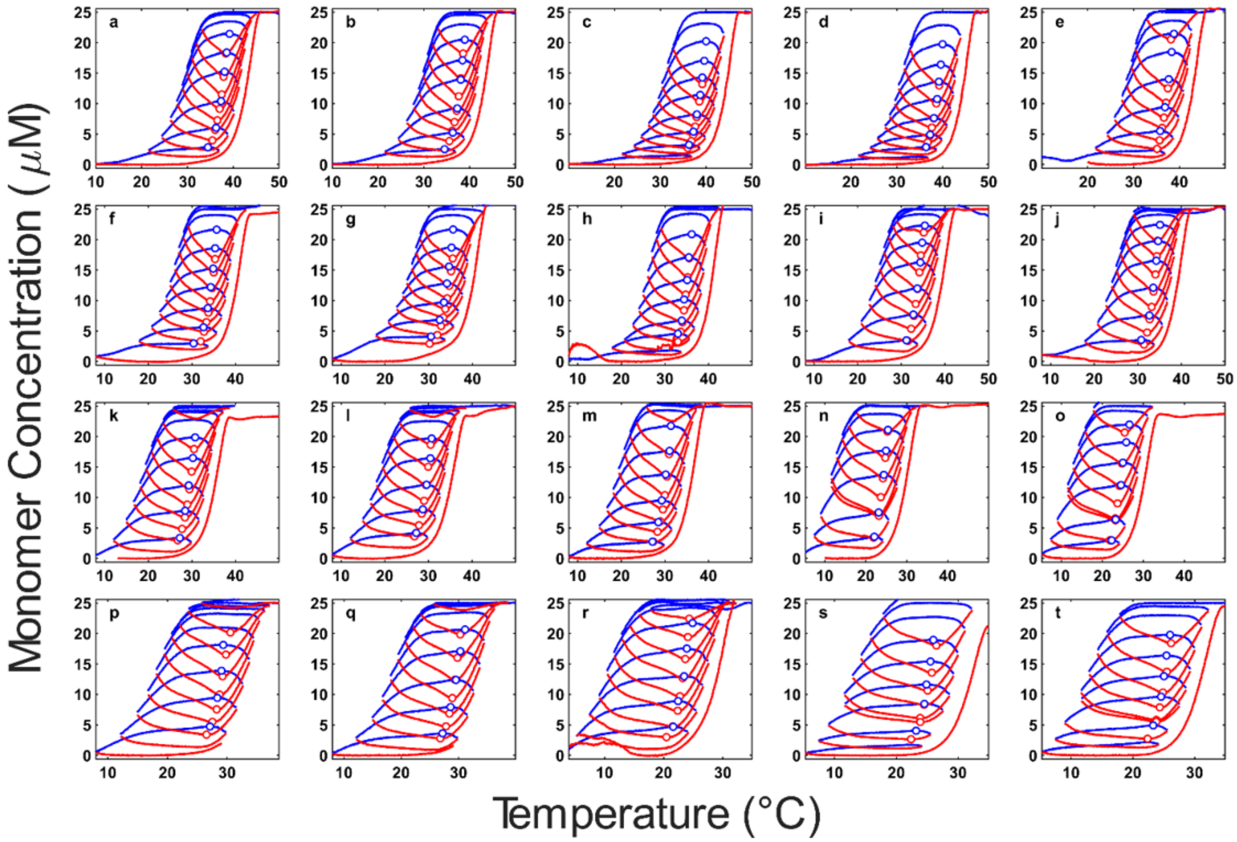

Figure S5: ***TREQ experiments of polyA-CA assembly.*** *At 15mM (a-d), 12.5mM (e-h), 10mM (i-l), and 7.5mM (m-p) cyanuric acid. Cooling traces are indicated in blue, heating traces are indicated in red. Extrema are shown as circles.*

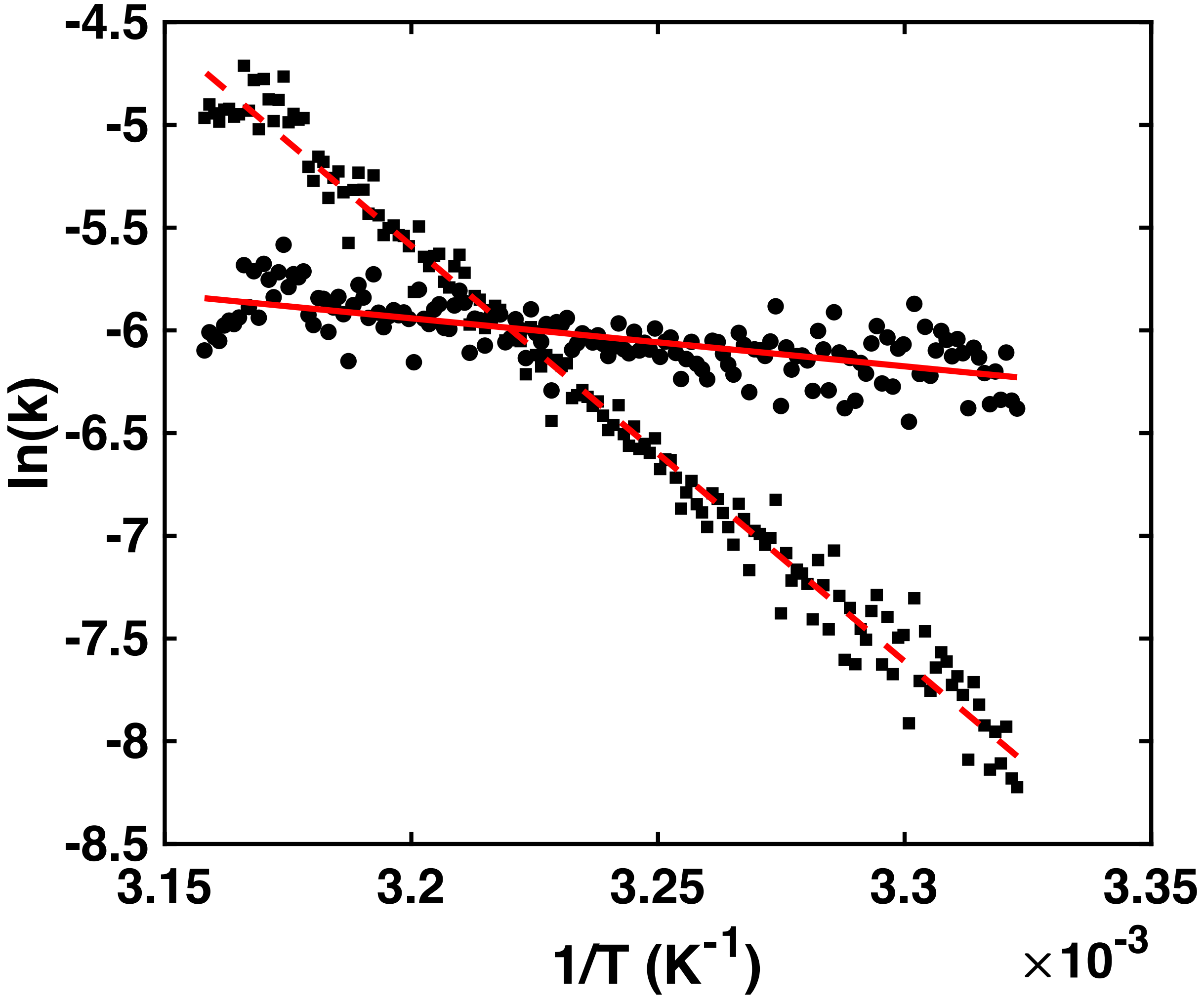

Figure S6: ***Arrhenius plots from classical two-state analysis of intramolecular G4 TH profiles taken at 0.1 K min^{-1}.*** *The analysis was applied to the portion of the curve between 0.15 < θ_U < 0.75. The folding (k_F) and unfolding (k_U) rate constants calculated from the experimental datasets are shown as circles and squares respectively, while the corresponding line of best fit to each dataset are shown as solid and dashed red lines respectively. The equilibrium melting temperatures are at the intersections of the folding and unfolding lines, and the equilibrium profile can be calculated from the ratio of k_F and k_U.(10)*

ΔH_e	99.5 ± 0.2 kcal mol^{-1}
ΔS_e	213.2 ± 0.5 cal mol^{-1} K^{-1}
ΔCp_e	-0.69 ± 0.02 kcal mol^{-1} K^{-1}
c_e	10.15 ± 0.08

Table S5: ***Thermodynamic parameters from a Van 't Hoff fit of the constant stoichiometry model in Figure 6 in the main text.*** *ΔS_e and ΔS_e are reported at a reference temperature of 25˚C.*

ΔH_{polyA}	-0.5 ± 0.5 kcal mol^{-1}
ΔS_{polyA}	8 ± 2 cal mol^{-1} K^{-1}
ΔH_{CA}	9.46 ± 0.05 kcal mol^{-1}
ΔS_{CA}	20.7 ± 0.2 cal mol^{-1} K^{-1}

Table S6: ***Thermodynamic parameters from Van 't Hoff fit of the independent sites model in Figure 6 in the main text.*** *The relatively large errors in ΔH_{polyA} and ΔS_{polyA} are caused by a correlation in the two parameters.*

Converting absorbance data to concentrations

The first step in analyzing TREQ data is converting the raw spectroscopic output into fractions of folding or assembly, or equivalently, concentrations of unfolded or unassembled monomers. Thermal melting and annealing data typically have linear regions at temperatures below and above the transition.(*10*) The linear data points are identified by eye and fitted by linear regression to obtain the slopes, m_L and m_U, and y-intercepts b_L and b_U of the lower and upper linear regions, respectively. The fraction folded or assembled, θ(T), is then calculated as

$$\theta(T) = \frac{(m_L T + b_L) - S_{exp}(T)}{(m_L T + b_L) - (m_U T + b_U)} \qquad (Equation\ S1)$$

where $S_{exp}(T)$ is the experimental absorbance (or fluorescence, or circular dichroism) measurement. The temperature-dependent monomer (M_1) concentration is calculated from θ(T) according to

$$[M_1](T) = \big(1 - \theta(T)\big)[M]_{tot} \qquad (Equation\ S2)$$

where $[M]_{tot}$ is the total concentration of monomers in all assembled forms.

Guide for acquisition of TREQ data

The design of a TREQ experiment involves selecting an appropriate scan rate and choosing a series of temperature set points that define the cooling and heating scans, for example (cooling $T_1 \rightarrow T_2$), (heating $T_2 \rightarrow T_3$), (cooling $T_3 \rightarrow T_4$), (heating $T_4 \rightarrow T_5$), etc. Slower scan rates lead to better-defined TREQ maxima and minima but longer experiments. We would suggest scan rates on the order of 0.2 to 1 °C/min. Slower assembly/disassembly kinetics require slower scan rates, although the success of the experiment is not particularly sensitive to the choice of scan rate. The selection of temperature set points is more critical as they will determine whether or not the system will pass through transient equilibria on the cooling and heating scans and generate a series of minima and maxima in the spectrophotometric data. We have developed a simple method for selecting the temperature set points that reliably produces high quality TREQ data. In the first step, a full cooling scan from maximum (T_{max}≈95°C) to minimum (T_{min}≈5°C) temperature is performed at the chosen scan rate, followed by a heating scan from T_{min} to T_{max}. The cooling scan will lie to substantially lower temperatures than the heating scan, due to thermal hysteresis, and the desired equilibrium curve lies somewhere between two. The temperatures at which the cooling scan generates 10, 20, 30, 40, etc. percent assembly are identified, yielding T_{C10}, T_{C20}, T_{C30}, T_{C40}, etc. respectively. The same analysis is performed for the heating scan, giving T_{H10}, T_{H20}, T_{H30}, T_{H40}, etc. We find that the oscillating cooling sequence $T_{max} \rightarrow T_{C10} \rightarrow T_{H10} \rightarrow T_{C20} \rightarrow T_{H20} \rightarrow \ldots \rightarrow T_{C90} \rightarrow T_{H90} \rightarrow T_{min} \rightarrow T_{max}$ reliably gives good quality TREQ data. The final heating scan is performed in order to obtain an adequate low temperature baseline. In principle, the oscillating heating sequence: $T_{min} \rightarrow T_{H90} \rightarrow T_{C90} \rightarrow T_{H80} \rightarrow T_{C80} \rightarrow \ldots \rightarrow T_{H10} \rightarrow T_{C10} \rightarrow$

$T_{max} \rightarrow T_{min}$ also produces similar TREQ data, however we prefer to begin each experiment with a fully thermally denatured sample for the sake of reproducibility (i.e. the first sequence). Note that we chose 10% increments in assembly because our spectrophotometer software allows up to 20 scans to programmed in advance.

Guide for processing TREQ data

The reliability of the TREQ experiments depends on accurately pinpointing the extrema of the scans, i.e. choosing the values of T_{ext}, $[M]_{ext}$, where $[M]_{ext}$ is the maximum or minimum value of [M] on each cooling or heating scan and T_{ext} is the temperature at which this is reached. Simply picking the maximum- or minimum-valued datapoint of each arc is inaccurate. Due to instrument noise, roughly half of the measured points lie above the true curve and the other half lie below. The point with the largest (or smallest) value will therefore almost certainly over (or under) estimate the true $[M]_{ext}$ value. Furthermore, the convex and concave cooling and heating arcs are fairly broad, meaning that the temperature at which the single largest- or smallest-valued point occurs is strongly influenced by the stochastic nature of the experimental noise and will almost certainly differ from T_{ext}. We have developed two different approaches at two different levels of computational difficulty for accurately identifying the extrema. The first is simply to calculate a sliding window average, selecting the extreme value of the average as $[M]_{ext}$ and the centre of the window as T_{ext}. The second is to smooth the experimental data by fitting a curve to the data points and identifying the extremum of the fitted curve as T_{ext}, $[M]_{ext}$. We prefer to use an empirical polynomial function for smoothing rather than a mechanistic (eg GS model) calculation, since we wish to apply the TREQ approach even to systems where the precise kinetic mechanism is unknown.

In order to test the accuracy of these methods, we generated synthetic noisy TREQ data based on the GS fibre assembly model (see below) for which the true maxima and minima were known and compared these values with the results of the sliding window and polynomial smoothing calculations. The simulated TREQ data are shown in *Figure S7a* with dashed lines indicating the true (error-free) curves and black circles indicating synthetic data, sampled at 0.3°C intervals with 1% random noise. We found that a rolling average of 9 to 12 data points gave extrema close the

true values. A van ‘t Hoff plot of the true data (dashed lines) and sliding average of ten points (purple circles) shows good agreement *(Figure S7b)*. We repeated the calculation 1,000 times with a resampled selection of the data points, and the resulting standard deviations of the extrema are shown as error bars.(*50*) Next, we fit the upper halves of cooling curves and lower halves of heating curves to polynomial functions of different orders. We found qualitatively that polynomials of orders of about 5 to 15 delivered the best performance. Polynomials of lower orders were not able to faithfully reproduce the overall shape of the data and higher orders began to overly mimic the simulated noise. Extrema taken from fitted 5th-order polynomials (green circles) align with the true values even more closely than the sliding averages (purple circles). Bootstrapped uncertainties in the extrema were low; error bars are smaller than the symbols used in *Figure S7b*. We conclude that the polynomial smoothing approach provides a more accurate extraction of the extrema, however the performance of the sliding average approach is satisfactory and is simpler to apply if using standard spreadsheet software to analyze data. We have used polynomial smoothing throughout.

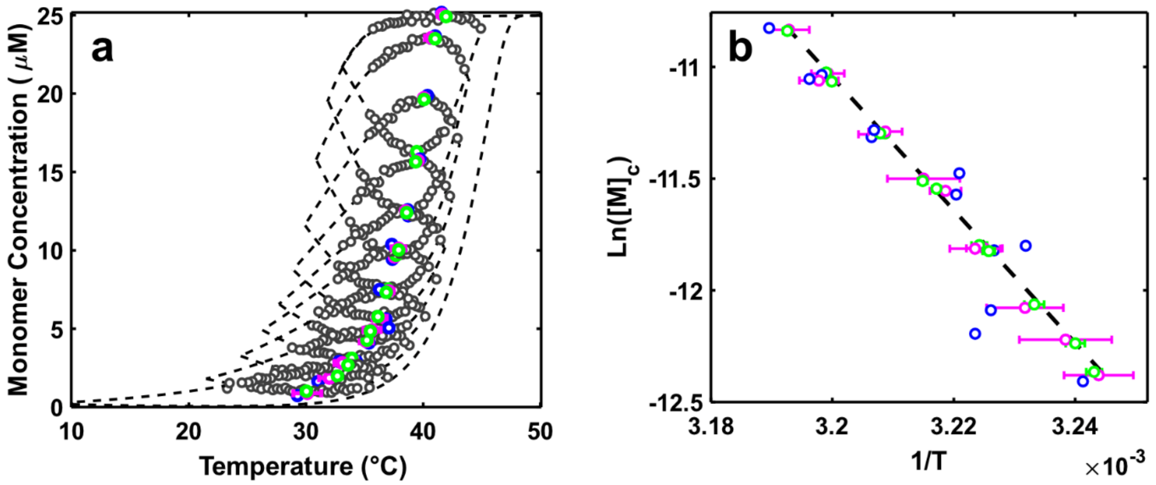

Figure S7: ***Analysis of simulated TREQ data with random noise.*** *a) Dashed lines represent a simulated TREQ data, grey circles represent the top or bottom half of each trace with added random noise. b) Van ‘t Hoff analysis of TREQ data, the dashed black line represents the true equilibrium curve. In both panels green circles represent extrema which were picked using a 5th order polynomial, magenta circles represent extrema which were picked using an averaging window of 10 data points and blue points represent extrema which were picked from raw data (i.e. max and min datapoints). Error bars in both monomer concentration and temperature are shown in both panels but are often smaller than the symbols.*

Thermodynamic analysis

TREQ data for polyA-CA fibres (critical polyA monomer concentrations, $[M]_c$, as a function of temperature, obtained at different CA concentrations) were fitted using two different physical models. In both cases, $[M]_c$ values were equated to the equilibrium dissociation constant for adding a monomer to the end of a growing fibre (K_e). The first model invoked constant CA:polyA stoichiometry (Equations 4-7 in the main text) and was essentially an extension of a classical van 't Hoff ln(K_e) vs 1/T analysis in which heat capacity changes and [CA] dependence are taken into account. The second model explicitly took into account the statistical effects of partially filling multiple binding sites (Master equations for high valence systems, Equation 9). In both cases, for each value of [CA], a $[M]_c(T)$ dataset was calculated in a temperature range from 10-50˚C with a resolution of 0.01˚C. Each model's parameters were optimized using total least squares regression, which accounts for errors in both x- and y- dimensions. Fits were optimized by finding thermodynamic parameters to minimize the target function

$$RSS = \sum_{j=1}^{N} \sqrt{\left(\frac{\Delta_{\ln([M]_{c(j)})}}{\sigma_{\ln([M]_{c(j)})}}\right)^2 + \left(\frac{\Delta_{\frac{1}{T_{(j)}}}}{\sigma_{\frac{1}{T_{(j)}}}}\right)^2}$$

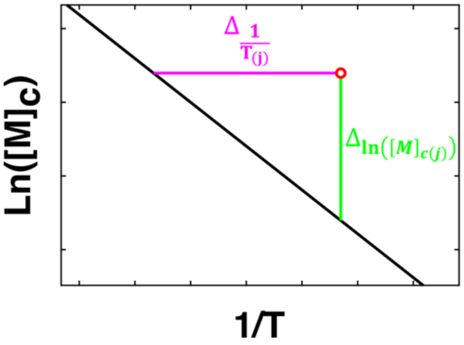

Figure S8: ***Minimization function for total-least squares regression and visualization of the horizontal (magenta) and vertical (green) distances of an experimental data point (red) to the simulated line (black).***

Where $\Delta_{\ln([M]_{c(j)})}$ is the vertical distance of the jth experimental data point to the point on the simulated curve which minimized the horizontal distance. $\Delta_{\frac{1}{T_{(j)}}}$ is the horizontal distance of the jth experimental point to the point on the simulated curve which minimized the vertical distance. $\sigma_{\ln([M]_{c(j)})}$ and $\sigma_{\frac{1}{T}(j)}$ are the experimental errors in the vertical and horizontal dimensions respectively. Errors for fitted parameters were calculated using a bootstrapping approach,(*50*) in

which each bootstrap sample was obtained by random resampling of the original data. For example, if the original dataset contained N points, each bootstrap sample was constructed by randomly selecting N of these data points, such that points may be selected more than once or not at all. 500 bootstrap samples were constructed and fitted using the thermodynamic models described above. The errors in the extracted parameters were taken as the standard deviations of the 500 sets of parameters obtained for all bootstrap samples.

Simplified analysis

An advantage of the TREQ approach is that rigorous analyses can be performed without specialized instrumentation, software, or expertise. The following is a quick guide for performing the characterization using standard spreadsheet software. First, typical thermal annealing/melting scans containing both a cooling and heating trace are run at the desired temperature scan rate for the TREQ experiment (typically 0.2-1˚C/min). These traces should be run over a large temperature range so that adequate linear baselines can be determined. These data should be converted to fraction assembled vs T, and the upper and lower temperatures selected for the TREQ experiment, as described above. The TREQ data are then converted to monomer concentration, $[M_1]$, vs T, as described above. The extrema of the arcs can be identified by the sliding window average, as described above (eg by using the *AVERAGE* function in Excel). Finally, care must be taken in the van 't Hoff analysis of $[M]_c$(T) datasets by linear regression of ln($[M]_c$) vs 1/T plots. Due to the flat shapes of many of the heating and cooling arcs, the relative error in the temperature dimension (x-axis) is substantially larger than that in the $[M_1]$ dimension (y-axis). Standard linear regression assumes that the uncertainty is entirely along the y-axis. Analyzing data with large errors in the abscissa variable leads to dilution bias in the slope estimates.(*51*) We have avoided this problem by performing total least squares regression, which takes into account different errors along the x- and y-dimensions.(*52*) A simpler approach to avoiding dilution bias is to perform traditional linear regression on an inverted plot of $\frac{1}{T}\ vs \ln([M]_c)$, since errors in 1/T are relatively much larger than those in ln($[M]_c$). The Enthalpy (ΔH) and Entropy (ΔS) can be calculated from the slope (m) and intercept (b) of this line as

$$\Delta H = -\frac{R}{m} \qquad (Equation\ S3)$$

$$\Delta S = \Delta H * b \qquad (Equation\ S4)$$

Generality of the Method

Our aim for the TREQ method is that it can be used as a general tool to determine the thermodynamic parameters of supramolecular assembly when standard thermal melting and annealing experiments are unsuitable for thermodynamic analysis. Towards this end, we have delineated criteria that enable the TREQ approach and have identified signatures in the data that indicate when these conditions are not met. We used computer simulations to model the TREQ experiment for different types of self assembling systems and observed two patterns of behaviour: either all the extrema aligned with the equilibrium curve or the maxima for the cooling curves and minima for the heating curves were offset from one another. This provides a useful guide for interpreting TREQ data on new systems of interest: when the extrema align, they can be used to trace out the equilibrium curve. When they are offset, they cannot be directly equated to equilibrium temperature/concentration pairs, although the data are still information-rich. Furthermore, when the extrema are offset, the system can be assumed to have violated one or both of two criteria outlined below. To proceed we make the following definitions: We will use species to refer to any set of assemblies that are kinetically and spectroscopically indistinguishable, and which may or may not be structurally identical. For instance, all GS fibres larger than the nucleus grow or shrink at the same rate and they can be collectively considered a single species, even though they comprise individual fibres of different lengths. The spectroscopic TREQ measurements report the concentration of just one species. This is referred to as the probed species, while all others are referred to as unprobed. Fast and slow chemical kinetics are defined relative to the temperature scan rate. The two TREQ criteria are 1) the effective rates at which the probed species interconverts with all other significantly populated species must be slow and 2) the effective rates at which all significantly populated unprobed species interconvert with each other must be fast.

For the polyA-CA fibres, there are only two significantly populated species: monomers (probed) and fibres larger than the nucleus (unprobed). Computer simulations of TREQ data show that the extrema align with the equilibrium curve. However, we have also investigated assembly

pathways that differ from the standard GS model. For instance, we previously studied the assembly of tetrameric guanine quadruplexes using thermal hysteresis.(*17*) The kinetics of assembly are consistent with a monomer ↔ dimer ↔ trimer ↔ tetramer pathway where only monomers (probed) and tetramers (unprobed) are significantly populated. Simulated TREQ data show that extrema closely follow the equilibrium curve *(Figure S9a)*, in good agreement with experimental data where the extrema align *(Figure S9c).* In contrast, if we consider the situation where dimers (unprobed) are also well populated and in fast exchange with monomers, criterion 1 is violated since the probed species exchanges rapidly with a well populated unprobed species. Simulated TREQ maxima and minima are now offset in this scenario *(Figure S9b)*. Finally, we studied a system that undergoes a parallel assembly mechanism. Tetra-amidated porphyrin molecules can assemble into either chiral fibres or achiral aggregates. As the temperature is reduced, the monomers first assemble into achiral aggregates that slowly convert to chiral fibres at low temperatures.(*53*) In this case, there are three well-populated species: chiral fibres (probed), achiral aggregates (unprobed), and monomers (unprobed). We performed a simulation in which achiral aggregates and monomers interconvert rapidly. This does not violate either criterion and the computed TREQ extrema align with the equilibrium curve *(Figure S9d)*. We then performed a simulation in which achiral fibres and monomers interconvert slowly, in violation of criterion 2, and the computed maxima and minima are offset from the equilibrium curve *(Figure S9e)*. Notably, this simulation closely matches experimental TREQ data for this system *(Figure S9f)* which shows the same pattern of offset extrema. Therefore, these data strongly suggest that interconversion between achiral fibres and monomers occurs slowly under these conditions and provide experimental validation for using offset extrema to identify situations that lie outside the scope of TE.

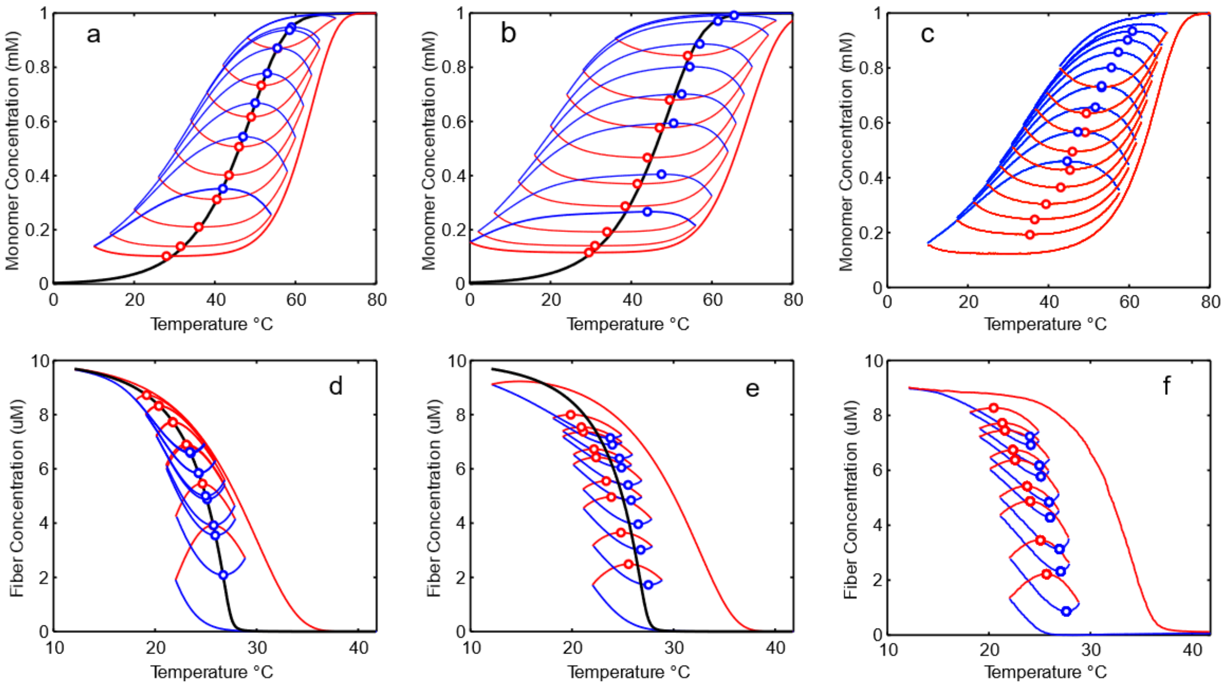

Figure S9: ***Simulated and experimental TREQ traces for different systems.*** *Top row) TREQ traces for sequential tetramolecular GQ assembly. a) Kinetic traces which have minimal kinetic intermediates. b) Kinetics which allow for build up of dimer intermediates. c) Experimental TREQ data showing that there are no kinetic intermediates. Bottom row) TEA traces for a zinc porphyrin system which has parallel pathways (one Isodesmic, one cooperative). d) A system with fast Isodesmic aggregation kinetics and slow cooperative aggregation kinetics. e) A system with slow Isodesmic aggregation kinetics and slow cooperative aggregation kinetics. f) Experimental TREQ trace showing a system which has slow Isodesmic aggregation kinetics and slow cooperative aggregation kinetics. Kinetic parameters for each simulation can be found in Table S7.*

Panel A				Panel B			
Activation energies		Rate constants		Activation energies		Rate constants	
E_1	-5.4	k_1	3.0×10^2	E_1	-5.4	k_1	3.0×10^2
E_{-1}	14.4	k_{-1}	5.0×10^3	E_{-1}	14.4	k_{-1}	5.0×10^3
E_2	-4.0	k_2	1.6×10^5	E_2	-4.0	k_2	2.0×10^4
E_{-2}	15.9	k_{-2}	3.1×10^{-1}	E_{-2}	15.9	k_{-2}	3.9×10^{-2}
E_3	-3.8	k_3	8.2×10^2	E_3	-3.8	k_3	8.2×10^2
E_{-3}	37.4	k_{-3}	8.4×10^{-3}	E_{-3}	37.4	k_{-3}	8.4×10^{-3}
		T_{ref}	45			T_{ref}	45
**Panel D**				**Panel E**			
**Activation energies**		**Rate constants**		**Activation energies**		**Rate constants**	
E_{n+}	-12.0	k_{n+}	6.0×10^7	E_{n+}	-12.0	k_{n+}	6.0×10^7
E_{n-}	6.0	k_{n-}	1.7×10^4	E_{n-}	6.0	k_{n-}	1.7×10^4
E_{e+}	-12.0	k_{e+}	6.0×10^7	E_{e+}	-12.0	k_{e+}	6.0×10^7
E_{e-}	12.4	k_{e-}	1.7	E_{e-}	12.4	k_{e-}	1.7
E_{i+}	-12.0	k_{i+}	3.0×10^6	E_{i+}	-12.0	k_{i+}	3.0×10^4
E_{i-}	1.0	k_{i-}	9.8	E_{i-}	1.0	k_{i-}	9.8×10^{-2}
		T_{ref}	25			T_{ref}	25

Table S7: ***Kinetic parameters for each TREQ simulation in Figure S9.*** *Activation energies are given in kcal mol^{-1} rate constants are in M^{-1} min^{-1} and min^{-1} for forward and reverse steps respectively. Reference temperatures are in °C.*

TREQ Simulations

TREQ experiments were simulated using the kinetic models described below. In all simulations the rate constants were assumed to have an Arrhenius temperature dependence following the equation

$$k(T) = k_0 e^{\frac{E_a}{R}\left(\frac{1}{T_{ref}}-\frac{1}{T}\right)} \qquad (Equation\ S5)$$

Each set of differential equations were numerically integrated as a function of temperature using MATLABs built in ODE solver ode15s. Temperature windows were chosen from experimental data windows, and kinetic parameters can be found in *Table S1, S2, S3, S4 and S7*.

TGGGG Assembly

Assembly of TGGGG strands into a guanine quadruplex was modelled as a sequential addition of monomers (M) into dimers (D), trimers (Tr) and tetramers (Q) using the following rate equations

$$\frac{d}{dt}[M] = 2k_{-1}[D] - 2k_1[M]^2 - k_2[M][D] + k_{-2}[Tr] - k_3[M][Tr] + k_{-3}[Q] \qquad (Equation\ S6)$$

$$\frac{d}{dt}[D] = k_1[M]^2 - k_{-1}[D] + k_{-2}[Tr] - k_2[M][D] \qquad (Equation\ S7)$$

$$\frac{d}{dt}[Tr] = k_2[M][D] - k_{-2}[Tr] + k_{-3}[Q] - k_3[M][Tr] \qquad (Equation\ S8)$$

$$\frac{d}{dt}[Q] = k_3[M][Tr] - k_{-3}[Q] \qquad (Equation\ S9)$$

polyA Assembly

polyA fibre formation was modelled following the Goldstein-Stryer model for cooperative self-assembly as described previously.(*32*) This model assumes reversible, cooperative stepwise addition of monomers (M) to nuclei (M_s), which then elongate to form fibres (M_N). The model has two distinct phases, where the pre-nucleus equilibria are governed by the nucleation rate constants k_{n+} and k_{n-}, and post-nucleus equilibria are governed by the elongation rate constants k_{e+} and k_{e-}. In order to limit the number of equations that must be numerically integrated, only fibres up to size N are explicitly described. A sparse Jacobian matrix was created to define the

species which are related, this allowed for simulations of large fibre sizes (N = 1000). The Goldstein-Stryer model is described by the following rate equations

Monomer

$$\frac{d}{dt}[M] = -k_{n+}[M]\left(2[M] + \sum_{i=2}^{s-1}[M_i]\right) - k_{e+}[M]\left(\sum_{i=s}^{N-1}[M_i]\right) + k_{n-}\left(2 * [M_2] + \sum_{i=3}^{s}[M_i]\right) + k_{e-}\sum_{i=s+1}^{N}[M_i] \qquad (Equation\ S10)$$

Pre-nucleus oligomers

$$\frac{d}{dt}[M_i] = k_{n+}[M]([M_{i-1}] - [M]) + k_{n-}([M_{i+1}] - [M_i]) \qquad (Equation\ S11)$$

Nucleus

$$\frac{d}{dt}[M_s] = k_{n+}[M][M_{s-1}] - k_{e+}[M][M_s] + k_{e-}[M_{s+1}] + k_{n-}[M_s] \qquad (Equation\ S12)$$

Post-nucleus fibres

$$\frac{d}{dt}[M_i] = k_{e+}[M]([M_{i-1}] - [M]) + k_{e-}([M_{i+1}] - [M_i]) \qquad (Equation\ S13)$$

Fibre length N

$$\frac{d}{dt}[M_i] = k_{e+}[M][M_{N-1}] - k_{e-}[M_N] \qquad (Equation\ S14)$$

Porphyrin Assembly

Zinc porphyrin assembly was modelled as a system with two distinct parallel pathways, where one pathway assembles via the Goldstein-Stryer model of assembly with pre-nucleated and post-nucleated rate constants of k_{n+}/k_{n-} and k_{e+}/k_{e-} up to a maximum length of N and a nucleus size s and one pathway forms Isodesmic aggregates which assembly with the rate constants k_{i+}/k_{i-} with a maximum length L. The parallel pathways model is described by the following rate equations.

Monomer

$$\frac{d}{dt}[M] = -k_{n+}[M]\left(2[M] + \sum_{i=2}^{s-1}[M_i]\right) - k_{e+}[M]\left(\sum_{i=s}^{N-1}[M_i]\right) + k_{n-}\left(2 * [M_2] + \sum_{i=3}^{s}[M_i]\right)$$
$$+k_{e-}\sum_{i=s+1}^{N}[M_i] - k_{i+}[M]\left(2[M] + \sum_{i=2}^{L-1}[I_i]\right) + k_{i-} * \left(2 * [I_2] + \sum_{i=3}^{L}[I_i]\right) \qquad (Equation\ S15)$$

Pre-nucleus oligomers

$$\frac{d}{dt}[M_i] = k_{n+}[M]([M_{i-1}] - [M]) + k_{n-}([M_{i+1}] - [M_i]) \qquad (Equation\ S16)$$

Nucleus

$$\frac{d}{dt}[M_s] = k_{n+}[M][M_{s-1}] - k_{e+}[M][M_s] + k_{e-}[M_{s+1}] + k_{n-}[M_s] \qquad (Equation\ S17)$$

Post-nucleus fibres

$$\frac{d}{dt}[M_i] = k_{e+}[M]([M_{i-1}] - [M]) + k_{e-}([M_{i+1}] - [M_i]) \qquad (Equation\ S18)$$

Fibre length N

$$\frac{d}{dt}[M_N] = k_{e+}[M][M_{N-1}] - k_{e-}[M_N] \qquad (Equation\ S19)$$

Isodesmic aggregates

$$\frac{d}{dt}[I_i] = k_{i+}[M]([I_{i-1}] - [M]) + k_{i-}([I_{i+1}] - [I_i]) \qquad (Equation\ S20)$$

Isodesmic aggregate length L

$$\frac{d}{dt}[I_L] = k_{i+}[M][I_{L-1}] - k_{i-}[I_L] \qquad (Equation\ S21)$$

REFERENCES AND NOTES

1. E. Busseron, Y. Ruff, E. Moulin, N. Giuseppone, Supramolecular self-assemblies as functional nanomaterials. *Nanoscale* **5**, 7098–7140 (2013).

2. J. A. Elemans, A. E. Rowan, R. J. Nolte, Mastering molecular matter. Supramolecular architectures by hierarchical self-assembly. *J. Mater. Chem.* **13**, 2661–2670 (2003).

3. P. T. Corbett, J. Leclaire, L. Vial, K. R. West, J.-L. Wietor, J. K. M. Sanders, S. Otto, Dynamic combinatorial chemistry. *Chem. Rev.* **106**, 3652–3711 (2006).

4. M. Medrano, M. A. N. Fuertes, A. Valbuena, P. J. Carrillo, A. Rodríguez-Huete, M. G. Mateu, Imaging and quantitation of a succession of transient intermediates reveal the reversible self-assembly pathway of a simple icosahedral virus capsid. *J. Am. Chem. Soc.* **138**, 15385–15396 (2016).

5. D. Pinotsi, A. K. Buell, C. Galvagnion, C. M. Dobson, G. S. Kaminski Schierle, C. F. Kaminski, Direct observation of heterogeneous amyloid fibril growth kinetics via two-color super-resolution microscopy. *Nano Lett.* **14**, 339–345 (2014).

6. E. Rennella, A. Sekhar, L. E. Kay, Self-assembly of human Profilin-1 detected by Carr–Purcell–Meiboom–Gill nuclear magnetic resonance (CPMG NMR) spectroscopy. *Biochemistry* **56**, 692–703 (2017).

7. M. Bellot, L. Bouteiller, Thermodynamic description of bis-urea self-assembly: Competition between two supramolecular polymers. *Langmuir* **24**, 14176–14182 (2008).

8. R. F. Pasternack, J. I. Goldsmith, S. Szép, E. J. Gibbs, A spectroscopic and thermodynamic study of porphyrin/DNA supramolecular assemblies. *Biophys. J.* **75**, 1024–1031 (1998).

9. T. F. De Greef, M. M. Smulders, M. Wolffs, A. P. Schenning, R. P. Sijbesma, E. Meijer, Supramolecular polymerization. *Chem. Rev.* **109**, 5687–5754 (2009).

10. J.-L. Mergny, L. Lacroix, Analysis of thermal melting curves. *Oligonucleotides* **13**, 515–537 (2003).

11. E. E. Greciano, S. Alsina, G. Ghosh, G. Fernández, L. Sánchez, Alkyl bridge length to bias the kinetics and stability of consecutive supramolecular polymerizations. *Small Methods* **4**, 1900715 (2020).

12. A. Osypenko, E. Moulin, O. Gavat, G. Fuks, M. Maaloum, M. A. J. Koenis, W. J. Buma, N. Giuseppone, Temperature control of sequential nucleation–growth mechanisms in hierarchical supramolecular polymers. *Chem. A Eur. J.* **25**, 13008–13016 (2019).

13. S. Singh, A. J. Zlotnick, Observed hysteresis of virus capsid disassembly is implicit in kinetic models of assembly. *J. Biol. Chem.* **278**, 18249–18255 (2003).

14. L. Sambe, V. R. de La Rosa, K. Belal, F. Stoffelbach, J. Lyskawa, F. Delattre, M. Bria, G. Cooke, R. Hoogenboom, P. Woisel, Programmable polymer-based supramolecular temperature sensor with a memory function. *Angew. Chem. Int. Ed.* **53**, 5044–5048 (2014).

15. K. Mizuno, S. P. Boudko, J. Engel, H. P. Bächinger, Kinetic hysteresis in collagen folding. *Biophys. J.* **98**, 3004–3014 (2010).

16. A. Dastan, W. J. Frith, D. J. Cleaver, Thermal hysteresis and seeding of twisted fibers formed by achiral discotic particles. *J. Phys. Chem. B.* **121**, 9920–9928 (2017).

17. R. W. Harkness, V. N. Avakyan, H. F. Sleiman, A. K. Mittermaier, Mapping the energy landscapes of supramolecular assembly by thermal hysteresis. *Nat. Commun.* **9**, 3152 (2018).

18. R. W. Harkness, C. Hennecker, J. T. Grün, A. Blümler, A. Heckel, H. Schwalbe, A. K. Mittermaier, Parallel reaction pathways accelerate folding of a guanine quadruplex. *Nucleic Acids Res.* **49**, 1247–1262 (2021).

19. M. Yamaguchi, Thermal hysteresis involving reversible self-catalytic reactions. *Acc. Chem. Res.*, 10226–10234 (2021).

20. T. Fukushima, K. Tamaki, A. Isobe, T. Hirose, N. Shimizu, H. Takagi, R. Haruki, S.-i. Adachi, M. J. Hollamby, S. Yagai, Diarylethene-powered light-induced folding of supramolecular polymers. *J. Am. Chem. Soc.* **143**, 5845–5854 (2021).

21. H. Kar, G. Ghosh, S. Ghosh, Solvent geometry regulated cooperative supramolecular polymerization. *Chem. A Eur. J.* **23**, 10536–10542 (2017).

22. Z. Fernández, B. Fernández, E. Quiñoá, F. Freire, The competitive aggregation pathway of an asymmetric chiral oligo(p-phenyleneethynylene) towards the formation of individual P and M supramolecular helical polymers. *Angew. Chem. Int. Ed.* **60**, 9919–9924 (2021).

23. F. Xu, L. Pfeifer, S. Crespi, F. K.-C. Leung, M. C. A. Stuart, S. J. Wezenberg, B. L. Feringa, From photoinduced supramolecular polymerization to responsive organogels. *J. Am. Chem. Soc.* **143**, 5990–5997 (2021).

24. S. Sarkar, A. Sarkar, S. J. George, Stereoselective seed-induced living supramolecular polymerization. *Angew. Chem. Int. Ed.* **59**, 19841–19845 (2020).

25. A. T. Haedler, S. C. J. Meskers, R. H. Zha, M. Kivala, H.-W. Schmidt, E. W. Meijer, Pathway complexity in the enantioselective self-assembly of functional carbonyl-bridged triarylamine trisamides. *J. Am. Chem. Soc.* **138**, 10539–10545 (2016).

26. S. Ogi, V. Stepanenko, K. Sugiyasu, M. Takeuchi, F. Würthner, Mechanism of self-assembly process and seeded supramolecular polymerization of perylene bisimide organogelator. *J. Am. Chem. Soc.* **137**, 3300–3307 (2015).

27. H. Wang, Y. Zhang, Y. Chen, H. Pan, X. Ren, Z. Chen, Living supramolecular polymerization of an Aza-BODIPY dye controlled by a hydrogen-bond-accepting triazole unit introduced by click chemistry. *Angewandte Chemie* **132**, 5223–5230 (2020).

28. R. D. Mukhopadhyay, A. Ajayaghosh, Living supramolecular polymerization. *Science* **349**, 241–242 (2015).

29. N. Avakyan, A. A. Greschner, F. Aldaye, C. J. Serpell, V. Toader, A. Petitjean, H. F. Sleiman, Reprogramming the assembly of unmodified DNA with a small molecule. *Nat. Chem.* **8**, 368–376 (2016).

30. A. Alenaizan, K. Fauché, R. Krishnamurthy, C. D. Sherrill, Noncovalent helicene structure between nucleic acids and cyanuric acid. *Chem. A Europn. J.* **27**, 4043–4052 (2021).

31. C. Lachance-Brais, C. D. Hennecker, A. Alenaizan, X. Luo, V. Toader, M. Taing, C. D. Sherrill, A. K. Mittermaier, H. F. Sleiman, Tuning DNA supramolecular polymers by the addition of small, functionalized nucleobase mimics. *J. Am. Chem. Soc.* **143**, 19824–19833 (2021).

32. R. F. Goldstein, L. Stryer, Cooperative polymerization reactions. Analytical approximations, numerical examples, and experimental strategy. *Biophys. J.* **50**, 583–599 (1986).

33. D. Zhao, J. S. Moore, Nucleation–elongation: A mechanism for cooperative supramolecular polymerization. *Org. Biomol. Chem.* **1**, 3471–3491 (2003).

34. P. Jonkheijm, P. van der Schoot, A. P. Schenning, E. Meijer, Probing the solvent-assisted nucleation pathway in chemical self-assembly. *Science* **313**, 80–83 (2006).

35. P. van der Schoot, *Supramolecular Polymers* (Taylor & Francis Group, 2005).

36. M. Arisawa, R. Iwamoto, M. Yamaguchi, Unstable and stable thermal hysteresis under thermal triangle waves. *ChemistrySelect* **6**, 4461–4465 (2021).

37. F. J. Rizzuto, C. M. Platnich, X. Luo, Y. Shen, M. D. Dore, C. Lachance-Brais, A. Guarné, G. Cosa, H. F. Sleiman, A dissipative pathway for the structural evolution of DNA fibres. *Nat. Chem.* **13**, 843–849 (2021).

38. R. W. Harkness, A. K. Mittermaier, G-register exchange dynamics in guanine quadruplexes. *Nucleic Acids Res.* **44**, 3481–3494 (2016).

39. N. R. Markham, M. Zuker, DINAMelt web server for nucleic acid melting prediction. *Nucleic Acids Res.* **33**, W577–W581 (2005).

40. P. J. Mikulecky, A. L. Feig, Heat capacity changes associated with nucleic acid folding. **82**, 38–58 (2006).

41. Self-processes—Programmed supramolecular systems, in *Supramolecular Chemistry* (1995), pp. 139–197.

42. J. J. M. Hamacek (Wiley Online Library, 2013), pp. 91–124.

43. A. P. Paneerselvam, S. S. Mishra, D. K. Chand, Linear and circular helicates: A brief review. *J. Chem. Sci.* **130**, 96 (2018).

44. J. Hamacek, M. Borkovec, C. Piguet, A simple thermodynamic model for quantitatively addressing cooperativity in multicomponent self-assembly processes—Part 1: Theoretical concepts and application to monometallic coordination complexes and bimetallic helicates possessing identical binding sites. *Chem. A Europn. J.* **11**, 5217–5226 (2005).

45. K. A. Dill, S. Bromberg, D. Stigter, *Molecular Driving Forces: Statistical Thermodynamics in Biology, Chemistry, Physics, and Nanoscience* (Garland Science, 2010).

46. Q. Li, J. Zhao, L. Liu, S. Jonchhe, F. J. Rizzuto, S. Mandal, H. He, S. Wei, H. F. Sleiman, H. Mao, A poly (thymine)–melamine duplex for the assembly of DNA nanomaterials. *Nat. Mater.* **19**, 1012–1018 (2020).

47. J.-M. Lehn, A. Rigault, J. Siegel, J. Harrowfield, B. Chevrier, D. Moras, Spontaneous assembly of double-stranded helicates from oligobipyridine ligands and copper (I) cations: Structure of an inorganic double helix. *Proc. Natl. Acad. Sci.* **84**, 2565–2569 (1987).

48. A. Mulder, J. Huskens, D. N. Reinhoudt, Multivalency in supramolecular chemistry and nanofabrication. *Org. Biomol. Chem.* **2**, 3409–3424 (2004).

49. M. Lin, Y. Dai, F. Xia, X. Zhang, Advances in non-covalent crosslinked polymer micelles for biomedical applications. *Mater. Sci. Eng. C* **119**, 111626 (2020).

50. B. Efron, R. Tibshirani, Bootstrap methods for standard errors, confidence intervals, and other measures of statistical accuracy. *Statist. Sci.* **1**, 54–75 (1986).

51. J. A. Hutcheon, A. Chiolero, J. A. Hanley, Random measurement error and regression dilution bias. *BMJ* **340**, c2289 (2010).

52. W. A. Fuller, *Measurement Error Models* (John Wiley & Sons, 2009), vol. 305.

53. R. van der Weegen, A. J. Teunissen, E. Meijer, Directing the self-assembly behaviour of porphyrin-based supramolecular systems. *Chem. A Europn. J.* **23**, 3773–3783 (2017).
